# Developmental Language Disorder as Syntactic Prediction Impairment

**DOI:** 10.3389/fcomm.2021.637585

**Published:** 2022-02-09

**Authors:** Arild Hestvik, Baila Epstein, Richard G. Schwartz, Valerie L. Shafer

**Affiliations:** 1 Department of Linguistics and Cognitive Science, University of Delaware, Newark, DE, United States,; 2 Communication Arts, Sciences, and Disorders, Brooklyn College, Boylan Hall, Brooklyn, NY, United States,; 3 PhD Program in Speech-Language-Hearing Sciences, The Graduate Center, City University of New York, New York, NY, United States

**Keywords:** syntax, gap-filling, prediction, event-related potentials, developmental language disorder, relative clauses, sentence processing

## Abstract

We provide evidence that children with Developmental Language Disorder (DLD) are impaired in predictive syntactic processing. In the current study, children listened passively to auditorily-presented sentences, where the critical condition included an unexpected “filled gap” in the direct object position of the relative clause verb. A filled gap is illustrated by the underlined phrase in “*The zebra that the hippo kissed the camel on the nose*…”, rather than the expected “*the zebra that the hippo kissed [e] on the nose*”, where [*e*] denotes the gap. Brain responses to the filled gap were compared to a control condition using adverb-relative clauses with identical substrings: “*The weekend that the hippo kissed the camel on the nose [e]*…”. Here, the same noun phrase is not unexpected because the adverb gap occurs later in the structure. We hypothesized that a filled gap would elicit a prediction error brain signal in the form of an early anterior negativity, as we have previously observed in adults. We found an early (bilateral) anterior negativity to the filled gap in a control group of children with Typical Development (TD), but the children with DLD exhibited no brain response to the filled gap during the same early time window. This suggests that children with DLD fail to predict that a relativized object should correspond to an empty position after the relative clause verb, suggesting an impairment in predictive processing. We discuss how this lack of a prediction error signal can interact with language acquisition and result in DLD.

## 1. INTRODUCTION

### 1.1 Syntactic Displacement

Displacement is the perturbation of syntactic constituents in the service of various speech acts, such as asking a question, focusing on something, restricting the meaning of the referent, passivizing a verb, topicalizing a constituent, and so on. It is an indispensable grammatical mechanism in human language. In relative clauses, such as “The man that Bill saw yesterday”, the relativized noun is related to a displaced direct object. During processing, a mechanism, called the parser, automatically generates a search for the origin of the displaced constituent, and generates predictions about where it will be found in the unfolding sentence structure (Crain and Fodor, 1985; but see Lewis and Vasishth, 2005; McElree, 2000 for alternative models). The current study examined whether children with Developmental Language Disorders (DLD) are impaired at predicting where the syntactic location of gaps should be, compared to their typically developing peers.

Several authors have observed that children with DLD are impaired in the use of Wh-questions (Deevy and Leonard, 2004; Marinis and van der Lely, 2007; Epstein et al., 2013) and relative clauses (Fonteneau and van der Lely, 2008; Friedmann and Novogrodsky, 2011; Hesketh, 2006; Hestvik et al., 2010; Schuele and Nicholls, 2000; Stavrakaki, 2001, 2002), and more generally with non-canonical word order (Montgomery and Evans, 2017). Different explanations for this have been offered in the literature, ranging from genetically caused impaired knowledge state (van der Lely and Pinker, 2014); impaired working memory resources (Weismer, 1996; Marton and Schwartz, 2003; Montgomery et al., 2017); slowed processing speed (Miller et al., 2001; Kail and Miller, 2006; Leonard et al., 2007), impaired sensory processing and speech perception leading ultimately to atypical morphosyntax and syntax (Leonard and Bortolini, 1998; Joanisse and Seidenberg, 2003), or impaired implicit learning (Evans et al., 2009; Plante et al., 2017). The aim of the current study is to investigate a previously unexplored possibility, namely that DLD has its root in prediction mechanisms (see Jones et al., 2021 for a recent discussion).

Prediction is increasingly recognized as a critical aspect of human cognition (Friston, 2005; Friston and Kiebel, 2009; Parr and Friston, 2018), and over the past decade, prediction has come to the forefront of psycholinguistic modeling and research (Levy, 2008; Rabagliati et al., 2016; Kuperberg and Jaeger, 2016; Gambi et al., 2018; Pickering and Gambi, 2018). Processing of filler-gap dependencies (the key component of relative clauses and Wh-questions) has long been known to involve predictions that arise from “active filler strategies” (Frazier and Fodor, 1978; Stowe, 1986; Stowe et al., 1991). We assume a model of filler-gap processing that includes the following assumptions: 1) An expression is recognized as a filler and is placed and maintained in working memory; 2) an “active” search for a gap position is initiated while the sentence representation is being incrementally built over time; 3) once a potential gap position is found, the filler is retrieved from memory and interpreted in this position—this is the step of “filling the gap” (Wagers and Phillips, 2013) or “antecedent reactivation” (Swinney et al., 1989; Love and Swinney, 1996). The active search stage involves predictions about how the sentence is likely to unfold; the parser predicts that it will encounter a position which can be interpreted as a gap in the sentence structure (Lau et al., 2006). This prediction in turn speeds up processing because predictions allow structure (and even lexical items) to be prebuilt before being encountered in the input stream. Pre-activation leads to faster integration of upcoming linguistic material (see Nieuwland and Kazanina 2020) for a recent review).

### 1.2 The Current Study: ERP Measure of Filler-Gap Processing in Developmental Language Disorders

Early work on gap-filling in typical populations focused on demonstrating that a filler is dynamically reactivated at the gap position, by using behavioral measures that tested for priming by the filler at the temporal juncture of the gap (Love and Swinney, 1996; Nicol et al., 2006). In Hestvik et al. (2010), we used a behavioral priming task with children with DLD, and found that they did not exhibit priming by the filler at the corresponding gap position, in contrast to a typical developing control group (see also Marinis and van der Lely 2007)). Using cross-modal priming, the control group of TD children exhibited priming at the gap position of stimuli related to the filler, but DLD children did not:

1. The zebra_*FILLER*_ that the hippo on the hill had kissed [*e*]_*GAP*_ on the nose ran far away

One possible explanation for lack of priming is that children with DLD, due to reduced working memory capacity (Weismer and Thordardottir, 1996; Fiebach et al., 2001; Marton and Schwartz, 2003; Montgomery, 2003; Ouchikh et al., 2016), are unable to maintain the filler in working memory (Sprouse et al., 2012; Kim et al., 2020), and are therefore slower at reactivating the filler at the gap position. If children with DLD are slower at reactivating the filler, perhaps at a delay after the verb, priming should be observed further downstream from the earliest possible gap-position. However, we have no model that predicts how much further downstream a gap might be postulated, making priming experiments impractical, as a 2×2 design is required at every hypothetical reactivation position. In addition, cross-modal priming tasks are cognitively demanding (e.g., using dual-task paradigms). Children with DLD may perform poorly on these behavioral tasks due to weaknesses in skills other than grammar, such as poor reading skills and or poor working memory capacity.

The goal of the current study was therefore to instead use a continuous measure of gap filling, via a study of predictive processing. ERPs exhibit millisecond timing of neural processes time-locked to a stimulus of interest and can provide an indication of the timing of a “surprise” response if a gap prediction is violated. The ERP technique is well-suited to test sentence processing in children and in language impaired populations. ERPs can be recorded to auditory sentences (thus, not requiring reading skills), and can use a relatively simple task (simple listening for comprehension). Despite the advantages of ERPs, only one study to date has used these measures to test gap-filling in children with DLD. Fonteneau and van der Lely (2008) presented sentences like (2) and time-locked the ERP to the underlined nouns:

- a. Who did Barbie push the clown into the wall?
  b. Who did Barbie push the ball into?

Their TD control group exhibited increased negativity over left anterior sites, within 300 ms of the onset of the “filled-gap” underlined noun phrase in (2a), compared to the direct object in (2b). In contrast, the children with DLD showed a later negativity that the authors interpreted as an N400 effect reflecting that the noun was processed as being semantically anomalous or unexpected, rather than ungrammatical. However, the study had several limitations. The wide age range of the participants (10–21 years of age) makes interpretation of the results difficult because considerable developmental differences in the timing and polarity of ERPs to syntactic violations have been observed (Hahne et al., 2004). Also, the study did not control for matching noun phrases in test and control conditions, and therefore the early ERP difference could reflect processing of different lexical items rather than detection of an unexpected grammatical form (Steinhauer and Drury, 2012).

In the current study, we used a “filled gap” paradigm that controls for lexical factors to measure the effect of prediction violations during sentence comprehension. We contrasted test sentences like (3a) with control sentences like (3b) (these materials were also used in studies with adults in Hestvik et al., 2007; Hestvik et al., 2012)):

- a. The zebra that the hippo kissed the camel on the nose ran far away.
  b. The weekend that the hippo kissed the camel on the nose, he ran far away.

**FIGURE 1 |:**
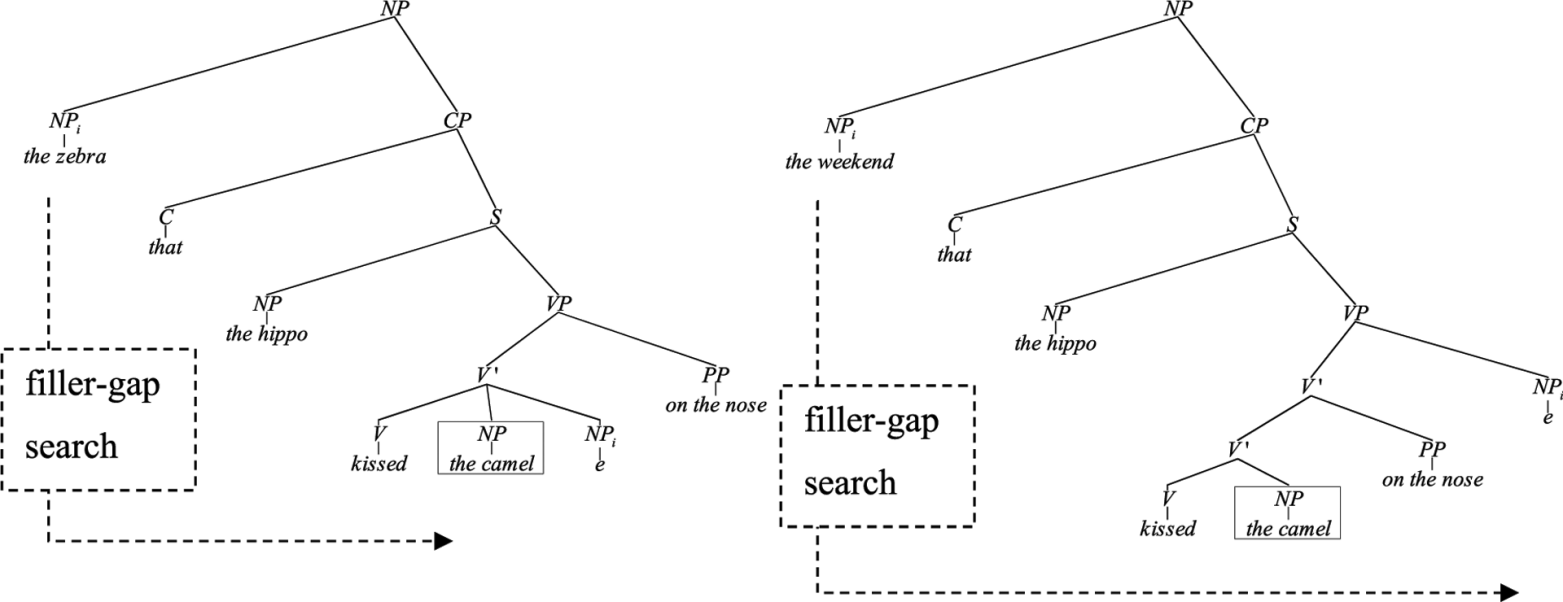
Parse trees for experimental (filled gap) and control condition stimuli sentences.

The only difference between sentence (3a) and (3b) is in the probability of encountering “the camel” immediately after the verb. The relativized noun phrase in (3a) is a direct object argument of the verb, which makes a post-verbal NP highly unexpected (and the sentence is ultimately ungrammatical). The control sentence (3b) is perfectly grammatical, as a time adverb has been relativized. The relativized adverb also leads to a search for its gap. However, the gap is located at the right periphery of the verb phrase, as illustrated in Figure 1 below; therefore, the occurrence of a noun phrase immediately following the verb is highly probable and not unexpected. Note that the two substrings and structures are otherwise identical. Thus, the only difference is in the grammatical function of the relativized noun, which predicts a direct object gap in (3a) but late adverb gap in (3b).

The experimental logic is illustrated in Figure 1. In both cases, we measured the brain response time-locked to the boxed NP “the camel”: If a surprise response is generated by ‘the camel’ in (3a) but not in (3b), the only source of this response is that a gap is predicted in place of the NP in (3a) and not in (3b).

We predicted that the surprise should be reflected by an early Left Anterior Negativity (Hahne and Friederici, 1999). This prediction was based on previous studies with adults, where filled gaps was found to elicit early left anterior negativity (~200 ms), LAN (400–500 ms), and P600 (Felser and Jessen, 2020; Hestvik et al., 2012, 2007). Our first study with adults using the current paradigm revealed an early left anterior negativity to the filled gap (Hestvik et al., 2007). In Hestvik et al. (2012) we observed an early bilateral anterior negativity (EAN) in the same paradigm. Bilateral anterior negativities to syntactic violations have been observed in other studies (Kessler et al., 2004; Pakulak and Neville, 2011). We view the eLAN and EAN as belonging to a family of syntactic violation ERP responses.

We also assume that the eLAN/EAN does not directly reflect ungrammaticality (Friederici (2012), but rather reflects probabilistic processing. This is because the filled-gap NP in Figure 1 does not make the sentence ungrammatical at the time point of its occurrence. The sentence could have a grammatical continuation, as in “The zebra that the hippo kissed the camel for.” Thus, the EAN here reflects a low probability syntactic category “event” rather than ungrammaticality. The eLAN/EAN has been observed in grammatical expectation violation studies with a wide range of languages (Neville et al., 1991; Münte et al., 1993; Rösler et al., 1993; Knosche et al., 1999; Hinojosa et al., 2003; Kubota et al., 2003, 2018; Ye et al., 2006; Brunelliere et al., 2007; Isel et al., 2007). Our design compares identical word strings and identical syntactic structures in test and control conditions leading up to the critical word, and therefore meets the design requirements for appropriate controls that previous studies have been criticized for (Steinhauer and Drury, 2012).

For children with DLD, we predicted an absent or delayed brain response to the filled gap. A delayed anterior negativity would be consistent with the hypothesis that children with DLD experience a “generalized slowing” (Miller et al., 2001; Montgomery, 2004; Leonard et al., 2007) but are otherwise unimpaired. An absent EAN to the filled gap would be consistent with a lack of predictive processing of filler-gap constructions, which could be the result of poor working memory capacity (Epstein et al., 2013) (but see Discussion below), or a lack of grammatical knowledge of filler-gap relations (van der Lely, 2005; van der Lely and Pinker, 2014).

## 2 METHODS

### 2.1 Participants

Thirty children (8–13 years) were recruited and enrolled in the study, which took place in Manhattan, New York. In accordance with the Helsinki Declaration, the study was approved by the Graduate Center CUNY Internal Review Board. All children provided informed assent, and their caretakers provided informed consent. Fourteen of the children met the criteria for DLD. Seventeen age-matched typically developing (TD) children served as the control group. One child with DLD was later diagnosed with ADHD and excluded from the analysis. Among the remaining 13 children with DLD, 5 were female and 8 male (matching the prevalence of higher incidence of DLD for boys than girls); and among the 17 children with TD, 7 were female and 10 were male. We used age-matching of the control group, because language-matching would have introduced age-related confounding effects (Plante et al., 1993).

Left-handers were not excluded from the study (2 participants), as about 70% of left-handers still have left-lateralized language, and language lateralization is not predictable from handedness (Knecht et al., 2000; Corballis, 2014; Somers et al., 2015). There is also little evidence that DLD is related to handedness (Bishop, 2013). In addition, a recent study found that left-handers did not differ from right-handers in the P600 index of morpho-syntactic violations (Grey et al., 2017).

The study was representative of the ethnic and racial diversity of New York City: 37% of all participants were Hispanic or Latino (55% in the DLD group); 40% of all participants where Black or African American (45% in the DLD group); one child with DLD was Asian. 41% of the TD group was Black or African American and the remainder were White. All children reported English as their first language, and all were from households where English was the primary language.

The children in the study passed a pure-tone hearing screening at 20 dB HL, based upon the guidelines of the American Speech-Language-Hearing Association (1997). The children in the DLD group were all receiving speech pathology services in school at the time of the study. None of the children in the study had any history of frank neurological impairments, psychological or emotional disorders, attention deficit disorders or other neuro-developmental disorders (as reported by parent questionnaires). The children in both groups (except one child in the TD group) were tested on a battery of tests: The Clinical Evaluation of Language Fundamentals (CELF-4, Semel et al., 2004), the Test of Nonverbal Intelligence (TONI-3, Brown et al., 1997) and the Peabody Picture Vocabulary Test (PPVT, Dunn and Dunn, 2007). Children with DLD scored at least 1.25 standard deviations below the mean on at least two of the four core subtests of the CELF-4. Table 1 provides means, standard deviations (SD) and ranges for these test scores and ages for each group. The mean expressive score on the CELF for the children in the DLD group was below 1.5 standard deviation of the population mean, but the mean PPVT score was within normal limits. Children in the TD group all scored within 1 SD of the mean on the CELF-4 and PPVT (see Table 1). Both groups of children scored within normal limits on the TONI-3.

As the descriptive statistics in Table 1 show, the groups are matched on age and age variance, as well as on the TONI, meeting the standard description of DLD as being within normal range on non-verbal IQ. The DLD participants differed from the reference population with effect sizes between 1.5 and 2 standard deviations for each language-specific test: The DLD means on the CELF-R, CELF-E and PPVT were 1.5. SD, 2.0 SD and 1.5 SD below the population means, respectively.

### 2.2 Materials

The within-subject independent variable contained two levels: Filled gap vs. control. In addition, three other sentence types were used in the experiment to reduce predictability of stimuli and to prevent the children from engaging in strategies to predict filled gaps. Sixty-four stimuli were constructed for each the five sentence types, illustrated in Table 2 (see the Supplementary Appendix for the full stimulus set).

#### 2.2.1 Comprehension Questions

A set of comprehension questions was constructed for each of the 64 stimulus sentences in the Filled Gap, Adjunct Control, Declarative and Object Relative sentence types. The comprehension questions served multiple purposes. The primary purpose was to ensure that participants paid attention to and computed the meaning of the stimulus sentences. A secondary purpose was to measure whether DLD children exhibited Sustained Negativity between the filler and the gap in object Wh-questions compared to subject Wh-questions; these results are reported in Epstein et al. (2013).

Finally, the comprehension questions were used to measure whether the DLD children differed from TD children in their understanding of the stimuli. There were four question types: Object Wh-questions (“Who did the alligator tap?”), subject Wh-questions (“Who bumped the duck?”), Yes-No questions (“Did the hippo kiss the camel?”) and a set of “easy” Yes-No questions (“Did you hear the word “road”?”). Question type was counterbalanced with the experimental condition type of the stimulus sentences (resulting in every question being asked four times over the entire experiment, but to different stimulus sentences). Thus, each subject heard 16 questions of each of the 4 question types, multiplied with 4 cells for a total of 256 questions. If DLD children failed to process sentences with filler-gap dependencies, they would be expected to exhibit guessing behavior for Adjunct Relatives and Object Relatives and should do worse on object Wh-questions than subject Wh-questions and Yes/No-questions, which do not involve long-distance dependencies.

To avoid asking comprehension questions after ungrammatical filled-gap sentences, each question was matched with two picture response options. One picture represented an object or character mentioned in the sentence. The other picture represented a question mark. Subjects were instructed to select the depicted object if it represented the answer, or alternatively the question mark if the depicted object did *not* represent the answer. Half the trials presented a picture depicting the correct answer, and the other half required choosing the *question mark* symbol. For the filled gap sentences, participants were expected to select the *question mark* response. This avoided asking a comprehension question to ungrammatical filled gap sentences.

Answers to comprehension questions were recorded by button press response and stored for analysis of accuracy and reaction time. An additional set of 38 “easy” filler sentences with heterogeneous structure (e.g., “The duckling and the chick that played near the barn ate all the seeds”) were followed not by question but instead exclamations like “Is that so?”, “You don’t say”, “Wow, ok”, “I like that”, and “That’s really nice,” so that not every sentence required a comprehension question (see the Supplementary Appendix for the full stimulus set).

#### 2.2.2 Audio-Recording of Stimuli

The stimulus sentences and questions were digitally recorded by a female speaker (16-bit resolution and 22050 Hz sampling rate), and the comprehension questions were recorded by a different female speaker. The speaker of the test sentences was a trained linguist, who consciously avoided giving prosodic cues about the presence of a gap. Two recordings were made of each pair and the best was selected for use. Acoustic analyses of the critical stimuli were conducted to determine whether they contained unintended duration or pitch cues to upcoming gap positions (Nagel et al.,1994). The mean and standard deviations of the durations from verb onset to the determiner “the” following the verb were virtually identical (M = 413 ms, SD = 71 for adjunct control vs. M = 414 ms, SD = 72 for filled gap), thus containing no prosodic cue to a gap. In addition, the pitch contours of the filled gap and control sentences were determined to be virtually identical, by visual inspection.

### 2.3 Procedure

Participants were fitted with a 64 channel Electrical Geodesics Sensor Net (v2) containing silver/silver-chloride (Ag/AgCl) plated electrodes encased in electrolyte-wetted sponges. One electrode was placed under each eye to monitor eye movements and eye blinks (see the Supplementary Appendix for the full spatial layout of the electrode montage).

Participants were seated in a sound- and electrically shielded audiometric booth (International Acoustics Co.) that was dimly lit. Participants faced a computer screen positioned at eye level at a 70 cm distance. The stimulus presentation was controlled by a PC with Psychology Software Tools (PST) E-Prime software (Schneider et al., 2002), and behavioral responses were collected with a PST Serial Response Box. The sentences and questions were presented at 65 dB SPL with two free-field loudspeakers, one placed behind and one directly in front of the subject. Participants were instructed to position the index and fourth finger of their right hand on the response box with labeled buttons. A single sentence trial proceeded as follows: First, a picture of an eye, serving as a fixation point and a reminder not to blink, appeared in the center of the computer screen for 100 ms. This was followed by auditory presentation of the stimulus sentence, with the fixation picture remaining on the screen during the presentation. After a 1,000-ms pause, participants heard the comprehension question. Two response options were depicted on the screen for a maximum of 7,000 ms. One button represented each depicted response option. Accuracy feedback was provided after each question, as well as the cumulative accuracy, to encourage participants to take the questions seriously and give them motivation to track and monitor their own performance. A 1,000-ms pause followed before the next trial.

Each participant began with a set of practice trials followed by all the stimuli in two consecutive sessions. Each session was divided into four blocks of 32 trials, randomly drawn from each of the sentence types. Short breaks were given between each block, and a longer break between the two sessions. Participants were told to listen to the sentences for meaning and answer the comprehension questions. The entire recording session took between 1½ and 2 h.

### 2.4 EEG Recording, Artifact Correction and Principal Component Analysis/Independent Component Analysis Preprocessing

**FIGURE 2 |:**
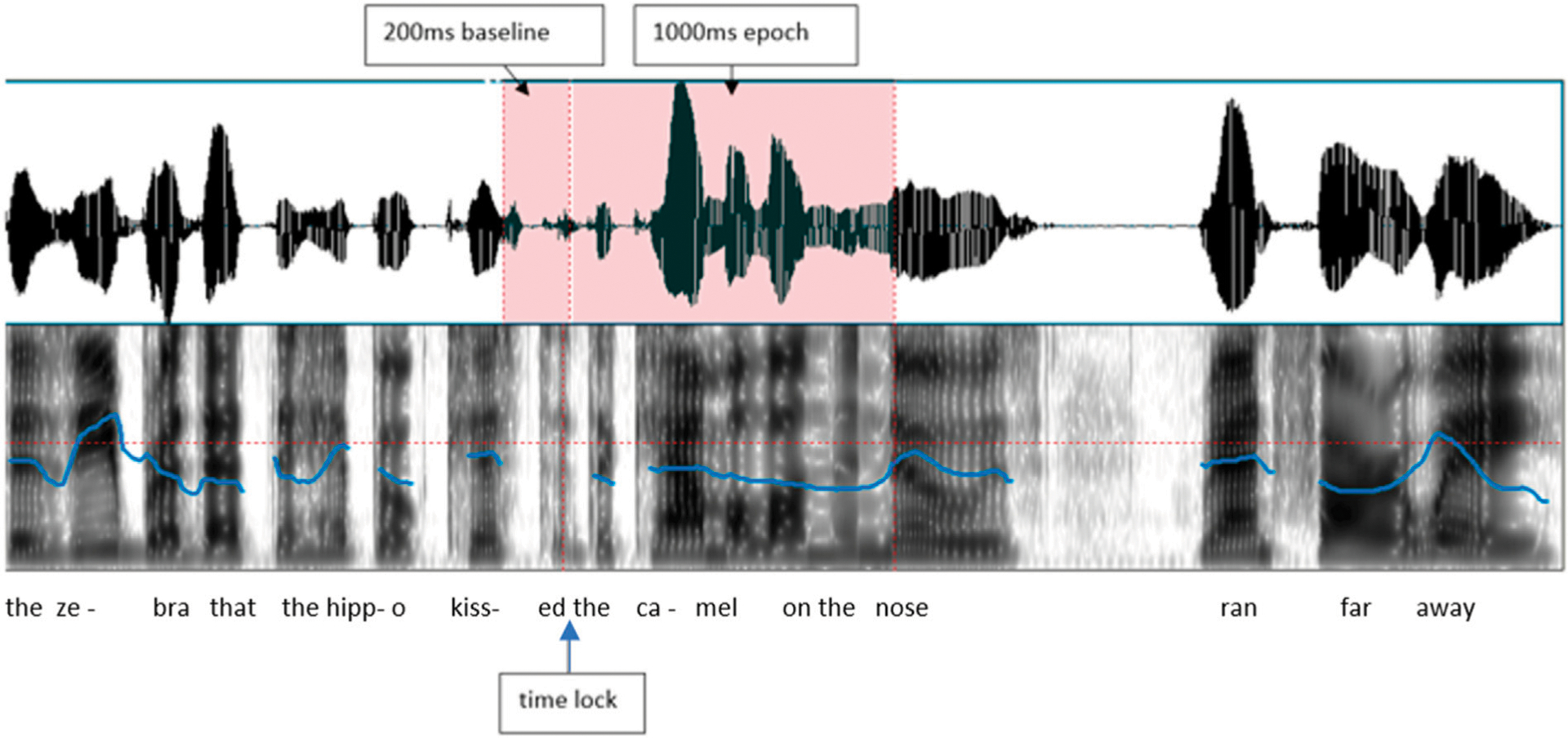
A stimulus sentence example, indicating where the EEG was measured from (between offset of verb and onset of the article “the”, and the baseline period.

EEG was recorded with an Electrical Geodesics, Inc. NetAmps 200 system. Electrode impedances were below 60 kOhm, acceptable for high impedance amplifiers (Ferree et al., 2001). EEG was sampled at 200 Hz, with Cz as the reference, a 0.1–41.2 Hz bandpass filter, and digitized with 12-bit resolution. Stimulus onset markers were placed by E-Prime between the offset of the verb and the onset of “the” (example: “…the hippo kissed [MARK] the camel…”). The continuous EEG was segmented into 1,200 ms epochs, including a 200 ms pre-stimulus baseline and a 1,000 ms epoch duration, using EGI Netstation Waveform Tools, as illustrated in Figure 2.

The epoched data were then submitted to a semi-automatic artifact detection procedure using Netstation software. A channel in a single recording was marked as a bad channel if the fast average amplitude exceeded 200 μV; if the differential amplitude exceeded 100 μV; or if it had zero variance. A channel was considered a bad channel in all trials if it was a bad channel on 20 percent of the trials. A trial was excluded if it contained more than 10 bad channels, or if it contained lateral eye movements resulting in amplitudes greater than ±70 μV. Bad channels were deleted and replaced with data from the surrounding electrodes using spherical spline interpolation, as long as those channels contained good data. All trials with eyeblink activity were removed. We chose this procedure as an alternative to subtracting eyeblink activity via independent component analysis (ICA) decomposition, as our experience is that ICA eyeblink subtraction distorts the anterior negativity ERP. This agrees with Luck (2014, p. 215) who cautions against use of ICA when the ERP overlaps with blink topography, which was the case in the current study. Trials were then baseline corrected by subtracting the mean voltage of the 200 ms baseline pre-stimulus period from the entire segment; trials were finally averaged across conditions for each subject. The data were then re-referenced to the average voltage (Luu and Ferree, 2005).

### 2.5 Behavioral Data Analysis Plan

The proportion of correct answers after the four sentence types (except the “easy” filler stimuli which had no questions) were analyzed with a mixed factorial repeated measures ANOVA with four levels of Stimulus Type: Adjunct relative clause (control), object relative clause with a filled gap (test), and as fillers, declarative, object relative clause (filler), and a 2-clause embedded declarative (filler). Crossed with this was the four levels of Question Type: object Wh-question, subject Wh-questions, yes/no questions and “easy” yes/no questions, with Group as the between-subjects variable. Before analysis, the mean proportion of correct answers to each cell of the question type (4) × stimulus sentence type (4) was transformed to logits (the natural logarithm of the odds of a proportion) to approximate the ANOVA requirement of continuous and normally distributed variables. We conducted separate ANOVAs with subject versus item as random factor (Clark, 1973).

### 2.6 EEG/ERP Analysis Strategy

In order to determine which time windows and electrode regions to analyze in the EEG data, we used Principal Component analysis (PCA) (Gorsuch, 1983; Spencer et al., 2001; Dien and Frishkoff, 2005; Dien, 2010, 2012) and ICA (Delorme and Makeig, 2004; Jung et al., 2001; Makeig et al., 1997, 2002). This approach reduces experimenter bias related to selecting electrode channels and time windows (Luck and Gaspelin, 2017) and reduces multiple comparison problems, as it delivers data-driven constructs of time-windows and electrode regions. This method allows a more objective means of identifying regions and time windows of interest than subjective visual inspection of the 65 sites and 250 time points per site.

A sequential PCA/ICA procedure (Dien and Frishkoff, 2005; Dien, 2010, 2012) was applied to extract the temporal and spatial dynamics of the EEG response to the experimental conditions, using the ERP PCA toolkit in MatLab (Dien, 2010). The PCA/ICA solution was then used to guide and constrain the selection of time windows and electrode regions for constructing dependent measures for ANOVA. We did plan to analyze an early time window over anterior sites, based on our previous studies, but the PCA/ICA analysis allowed for an objective method in calculating this temporal-spatial component. As a first step in the analysis, the mean difference waves (filled gap minus control) per subject served as input to a temporal PCA using the covariance matrix and *promax* rotation (k = 3) with Kaiser loading weighting (Hendrickson and White, 1964; Richman, 1986; Tataryn et al., 1999). Following this step, temporal components were retained that accounted for at least 5% of the variance and fulfilled the Parallel Test and Scree Test (Horn, 1965). A spatial PCA was then conducted on each retained temporal factor using the INFOMAX rotation on the covariance matrix i.e., ICA (Bell and Sejnowski, 1995). Spatial factors for each of these temporal factors that accounted for at least 1% of the variance were then examined to determine which components best matched the temporal-spatial pattern of the AN. Note that the amount of variance accounted for by spatial factors is not relevant in determining the importance of a factor, because more focal effects will necessarily account for less variance than a more broadly distributed effect (which will be spread across more electrode sites). We refer the reader to tutorials for further explanation of the PCA approach (Dien, 2010, Dien, 2012, Dien, 2020).

The factors identified in the PCA/ICA that matched the temporal-spatial properties of AN (early in time, with anterior negativity) and their associated factor scores were assessed for significance by being used as dependent measures in mixed factorial repeated measures ANOVA, with group as a between-subjects variable (conducted separately for each of the five factors). Since the PCA/ICA factors were derived from difference waves, a significant intercept is analogous to a main effect of condition; and a main effect of group is analogous to an interaction between group and condition. The undecomposed, unweighted voltage data was then analyzed by using the temporo-spatial PCA/ICA region to select a voltage for each subject, condition and trial and analyzed with inferential statistics. Here, we used a linear mixed model, accounting for both subject and item variance. The analyses were carried out using Statistica (Statistica, 2017) and *lme4* (Bates et al., 2015) R (R Core Team, 2017) software.

## 3 RESULTS

### 3.1 Behavioral Comprehension Data

In the subject-as-random factor analysis, the independent variables were question type (4 levels), and stimulus sentence type (4 levels), with question type crossed with stimulus sentence type. There were 16 unique questions in each type: object Wh-questions, subject Wh-questions, yes/no-questions and “easy” yes/no-questions. Each question was posed once in each of the four stimulus conditions, resulting in a 4 × 4 within-subject design. This ANOVA resulted in main effects of group, question type, and stimulus sentence type but no interactions involving group. The main effect of group was caused by TD children having overall higher accuracy than DLD children (71 vs. 62%), *F*(1,28) = 7.59, *p* = 0.01). The main effect of question type (F(3,84) = 111, *p* < 0.0001) was due to Object Wh-questions being the hardest (54% accuracy), followed by Subject Questions (63%) and Yes/No-questions (67%), with the “easy” Yes/No-questions (“did hear the word X”) having the highest accuracy (85%). There was no interaction between Question type and Group. There was also a main effect of stimulus sentence type, *F*(3,84) = 8.8, *p* < 0.001, such that the Adjunct control and relative clause filler had higher accuracy than the filled gap stimuli and the declarative clause fillers. Again, there was no interaction between Group and stimulus sentence type.

For the analysis with item (comprehension question) as random factor, the same 16 questions in each question type are now viewed as random samples of the infinite number of questions that could be formed within each type. The question type therefore becomes a grouping variable for questions (i.e., Wh-question, Yes/No-questions) and is in effect a “between-item” or grouping variable, with questions as the randomly sampled items that are being tested. Finally, participant group was added as a “within-item” variable for questions, because each question is tested repeatedly in both TD children and DLD children. The by-item analysis converged with the subject-as-random factor analysis in showing a main effect of group, and a main effect of question type and stimulus type. It differed from subject as random factor by exhibiting an interaction between question type and group. Inspection of the interaction plots revealed that this was driven by the “easy” Yes/No-questions (“did you hear the word “zebra”?”) having higher accuracy in the TD group.

**FIGURE 3 |:**
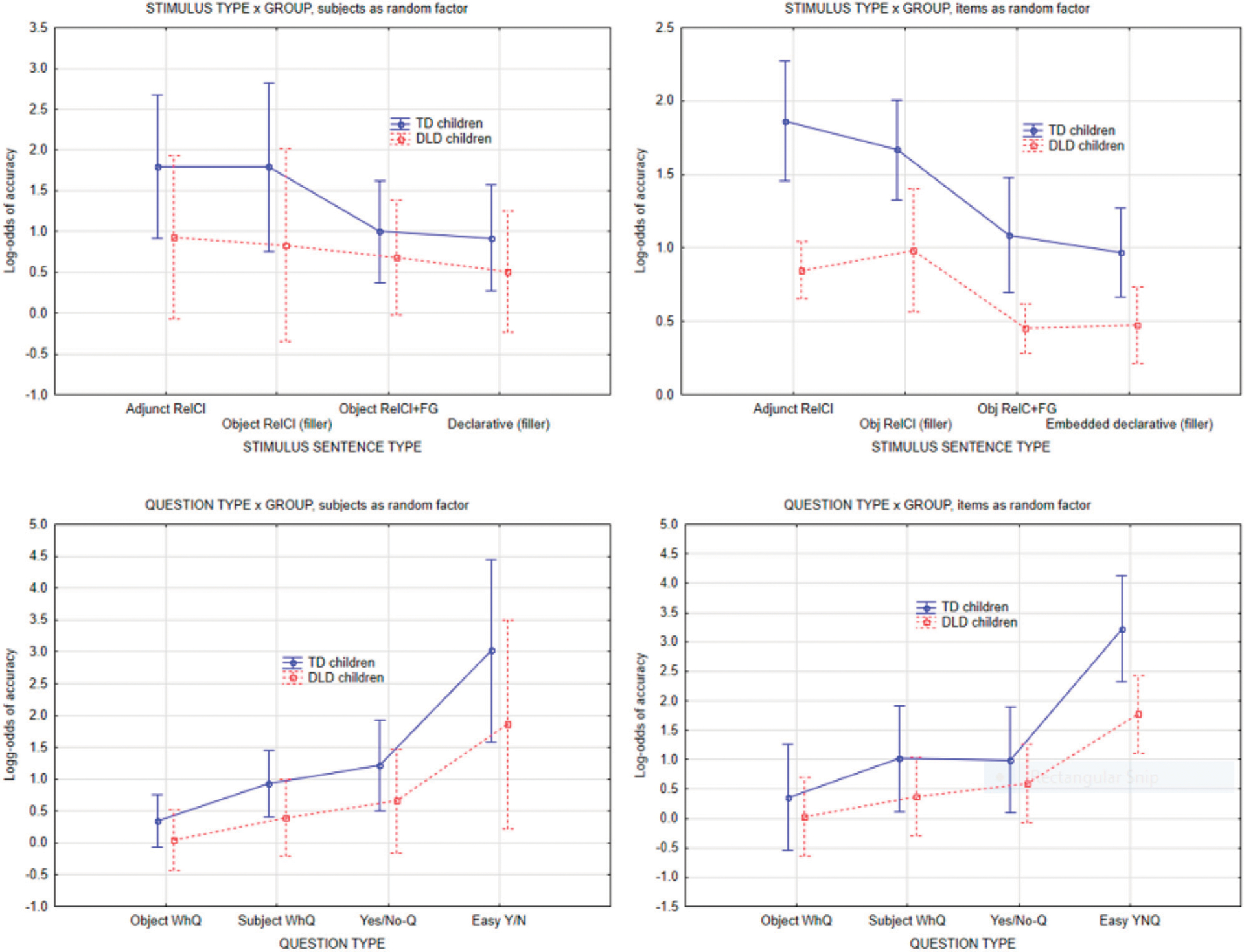
Accuracy on comprehension questions by question type and stimulus sentence type, subject as random factor vs. item as random factor. Error bars indicate 95% confidence intervals.

As Figure 3 shows, both groups of children exhibited a similar pattern of accuracy. There was a main effect of group such that TD children had higher accuracy, but there was no interaction between group and question type or stimulus type, indicating that accuracy was not grammatically conditioned (see Discussion).

### 3.2 ERP Results

After artifact detection and correction, the mean proportion of good trials in the two experimental conditions for the TD group was 55% (SD = 19%, range: 18–84%), and 56% for the DLD group (SD = 18%, range: 35–99%). In terms of actual numbers of trials per condition, the TD group averaged 35 trials (SD = 12) for the control condition and 36 trials (SD = 12) for the filled gap condition. For the DLD group, the average was 35 trials (SD = 14) for the control condition and 35 trials (SD = 14) for the filled gap condition. Thus, the groups were descriptively similar in terms of how many trials were included per condition.

As stated in the Methods section, we chose to remove trials with eyeblinks, rather than using ICA to subtract blink activity. The current study started out with 64 delivered trials per condition, twice as many as in Hestvik et al. (2007); therefore, the remaining trial count after blinks were removed was still fairly high for this kind of experiment. Although some participants in each group still had a relatively low trial count in each cell, we decided to keep all participants due to the difficulty of finding and recruiting children with DLD; cf. Faul et al. (2007) who point out that one must compromise between single-subject statistical power and being able to serve clinical populations.

#### 3.2.1 Descriptive ERP Results

**FIGURE 4 |:**
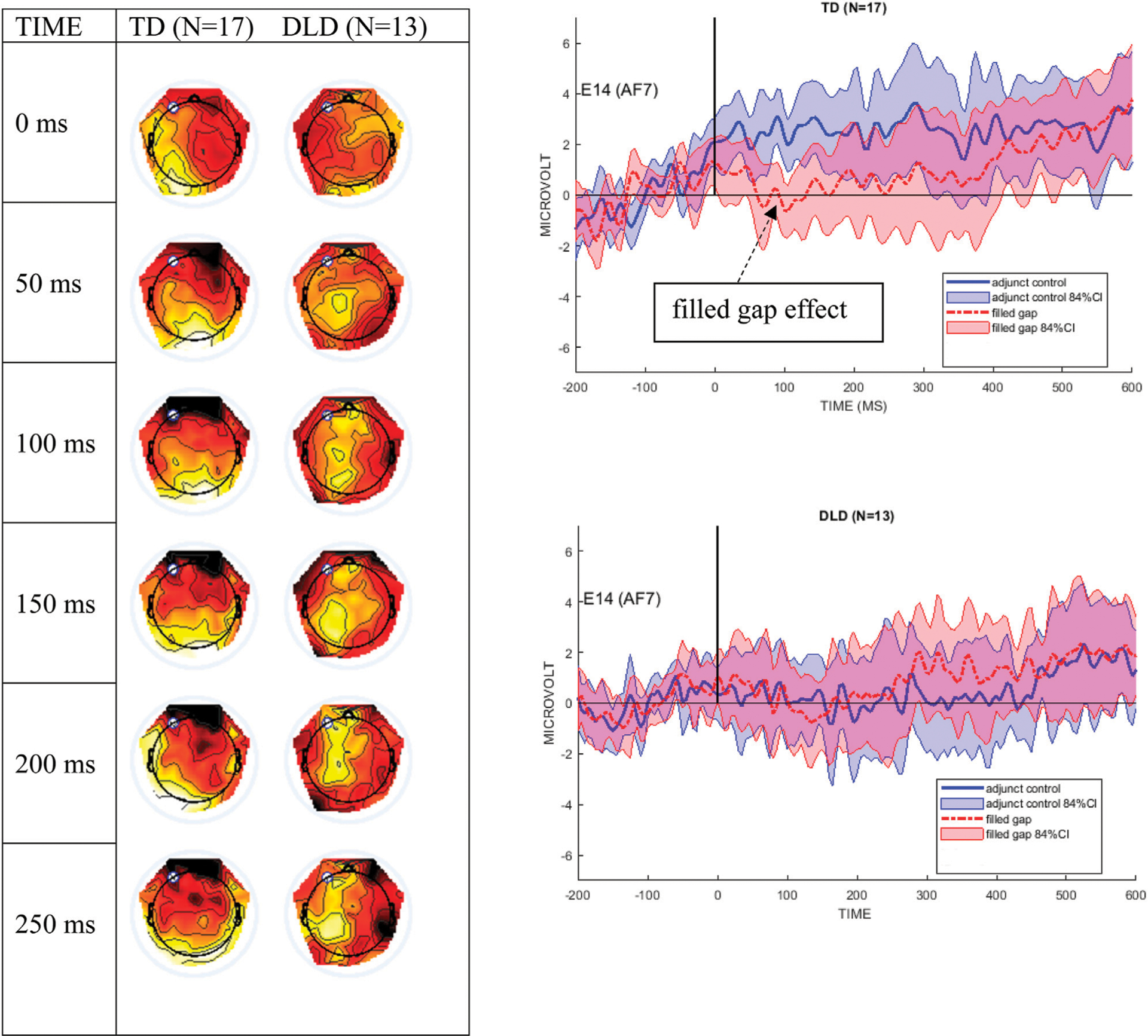
The left panel shows difference wave topographical plots at 50-ms increments from onset of the filled gap NP, for the TD and the DLD groups. Scale for color min/max: −2 μV (dark red) to +2 μV (white). The white dot is electrode E14 (AF7). The right panel shows electrode E14 for the TD and the DLD group, with 84% confidence interval bands around the filled gap vs. the adjunct control waves. We here follow authors who argue that 95% CIs are too conservative for ERP designs (Schenker and Gentleman, 2001; Payton et al., 2003; Dien, 2020).

Figure 4 shows the mean ERPs at Electrode site E14 (left anterior, near AF7 in the 10–10 system) and 84% confidence intervals (CIs) around the filled gap and control conditions. These graphs clearly show that the TD control group exhibited an early AN to the filled gap, between approximately 80 and 120 ms. In contrast, the DLD group showed no difference between conditions during this early time window. The left graph in Figure 4 shows the difference wave topography (ERPs to the filled gap minus control) at 50-ms intervals from stimulus onset for both groups. AF7 was the site where the largest effect was observed in previous studies reporting eLAN in a time range of 100–200 ms (Friederici et al., 1993; Hahne and Friederici, 1999), and was therefore chosen to illustrate the effect as waveforms (right panels). As shown, the confidence intervals separate conditions during the eLAN time window, suggesting a meaningful difference.

**FIGURE 5 |:**
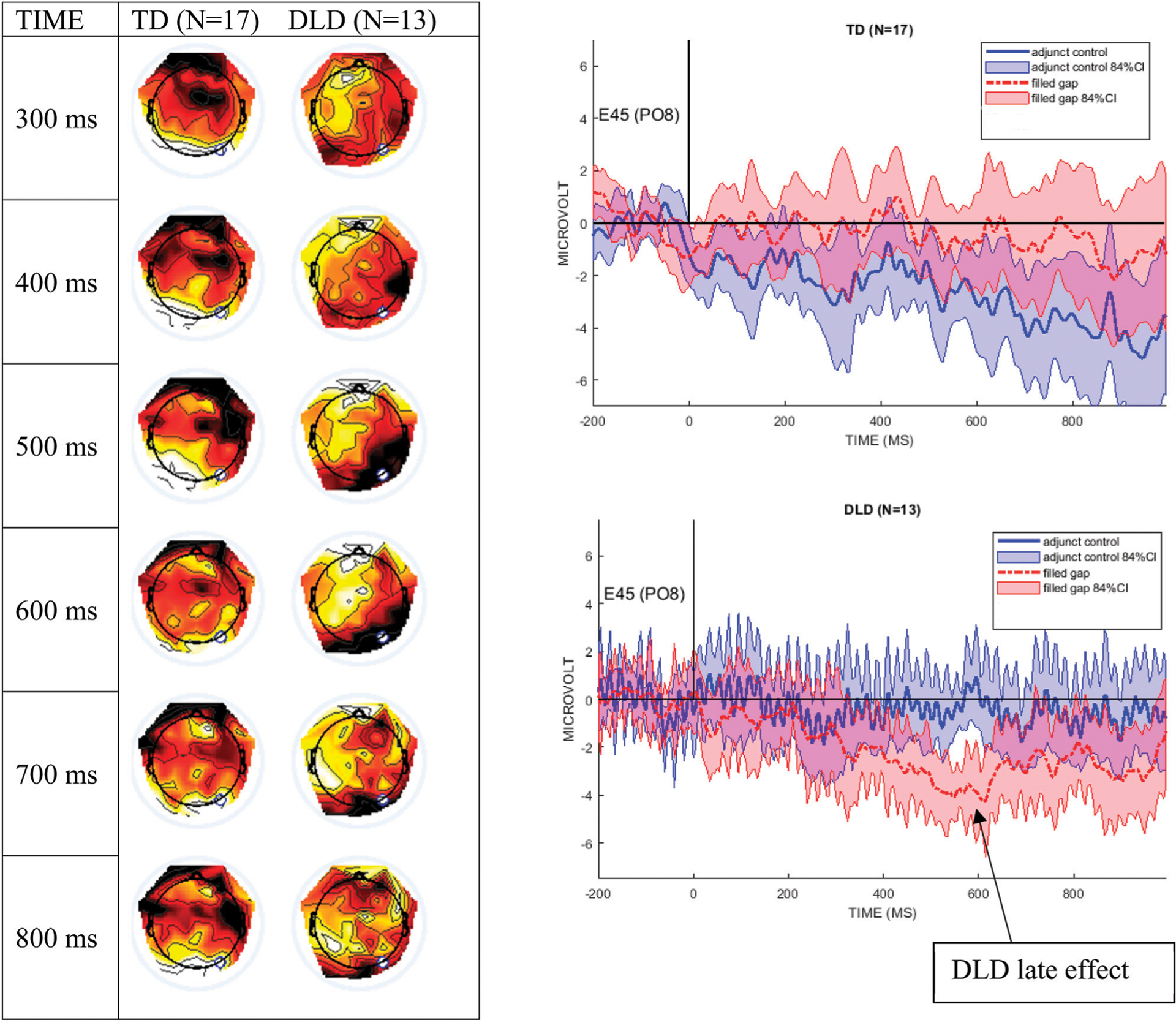
The left panel shows difference wave topographical plots at 100-ms increments between 300 and 800 ms from the onset of the filled gap NP, for the TD controls and the DLD group. Scale for color min/max: −2 μV (dark red) to +2 μV (white). The white dot is electrode E45 (PO8). The right panel shows electrode E45 for the DLD and the TD group, with 85% CI around the filled gap (red) vs. the adjunct control (blue) waves.

The DLD group shows an apparent late condition effect from 500–700 ms after stimulus onset. This pattern was characterized by an anterior positivity/right-posterior negativity and is shown in the difference topographical plots in Figure 5; the right panel graphs display the mean waveforms at electrode E45 (PO8 in the 10–10 system) with 84% CIs, revealing the greatest difference between conditions from 500–600 ms.

#### 3.2.2 Temporo-Spatial Principal Component Analysis/Independent Component Analysis Analysis

**FIGURE 6 |:**
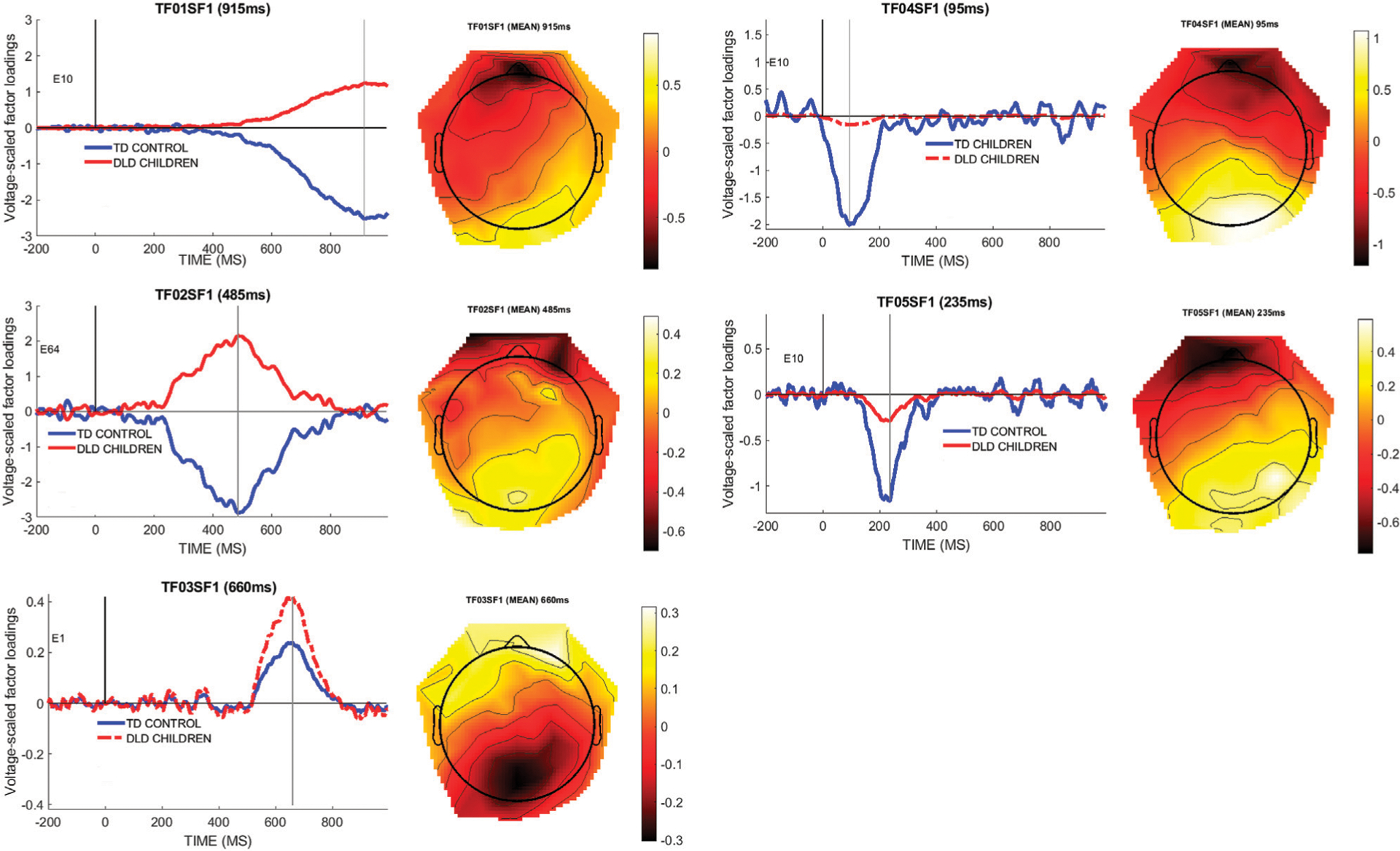
Temporal factors (peak latency indicated) and their spatial distribution; left panels show the microvolt-scaled factor loading waveforms by group. The right panels show the spatial distribution of the effects by using the mean (main effect) spatial component. Note that groups only differ from each other in the amplitude domain for the temporo-spatial factor so the spatial distribution of the factor is identical for all participants, as the PCA/ICA analysis is conducted on the pooled data. The second vertical line indicates the peak latency of the temporal component.

To determine an objective measure of the temporal and spatial dynamics of the brain response to the filled gap, we first conducted a PCA decomposition of the effects, as outlined in the Methods section. The temporal PCA of the difference wave (filled gap minus control) resulted in five retained temporal factors for further analysis, based on the criterion of selecting factors that accounted for at least 5% variance. Temporal factor 1 (TF1, peaking at 915 ms) accounted for 40% of the variance; TF2 (485 ms) accounted for 23% of the variance, TF3 (660 ms) accounted for 7%, TF4 (95 ms) accounted for 5% of the variance, and TF5 (235 ms) accounted for 5% of the variance. The spatial PCA step resulted in retaining 5 spatial factors for each temporal factor. The first spatial subfactor in each temporal factor accounted for most of the spatial variance (TF1SF1: 9.8%; TF2SF1: 5.1%; TF3SF1: 2.2%; TF4SF1: 1.6%; TF5SF1: 1.4%). The combined temporo-spatial factors accounted for 63% of the total variance in the data. Figure 6 below shows the five temporal/spatial components and the peak channel for the difference wave factors for each group, and a topographical plot for the main effect difference wave at the peak latencies.

As shown in Figure 6, four of the five factors exhibited an anterior negativity/posterior positivity pattern, from an early time window (TF04SF1, 95 ms) to a late time window (TF01SF1, 915 ms). (We performed the analysis also with linked mastoids as the reference, which did not affect the overall results.) The anterior negativity topographies were strongly driven by the TD control group of children, as can be seen in the figures. In contrast, a late factor (TF03SF1, 660 ms peak latency) exhibited the opposite polarity pattern and was more strongly driven by the DLD group of children. As we will interpret TF04SF1 as the early anterior negativity response to a syntactic category violation, we will henceforth label it as the “EAN (TF04SF1)” component, to differentiate it from the corresponding voltage component “EAN (voltage-ERP)” derived from this temporo-spatial factor (see below). The reason for this ambiguous denotation is that the PCA component and the voltage ERP represents two different approaches to analyze the same effect in the data.

Preliminary ANOVAS were performed separately for each of the five factors to determine their significance; using the mean factor scores per subject as input, the dependent variable was the factor score for the difference wave used as input to the PCA. Only TF04SF1 (95 ms), and no other temporo-spatial component, exhibited a statistically significant effect of group (*F*(1,28) = 4.99, *p* = 0.03, η^2^ = 0.15. In orthogonal contrast analysis comparing each group mean against zero, using dummy coding (1 for the group to be tested, 0 for the group to leave out), the difference wave was significantly different from 0 for the TD group (estimate −2.27, t = −3.03, *p* = 0.005), but not for the DLD group (estimate = 0.377, *t* = 0.411, *p* = 0.68), thus explaining the interaction.

**FIGURE 7 |:**
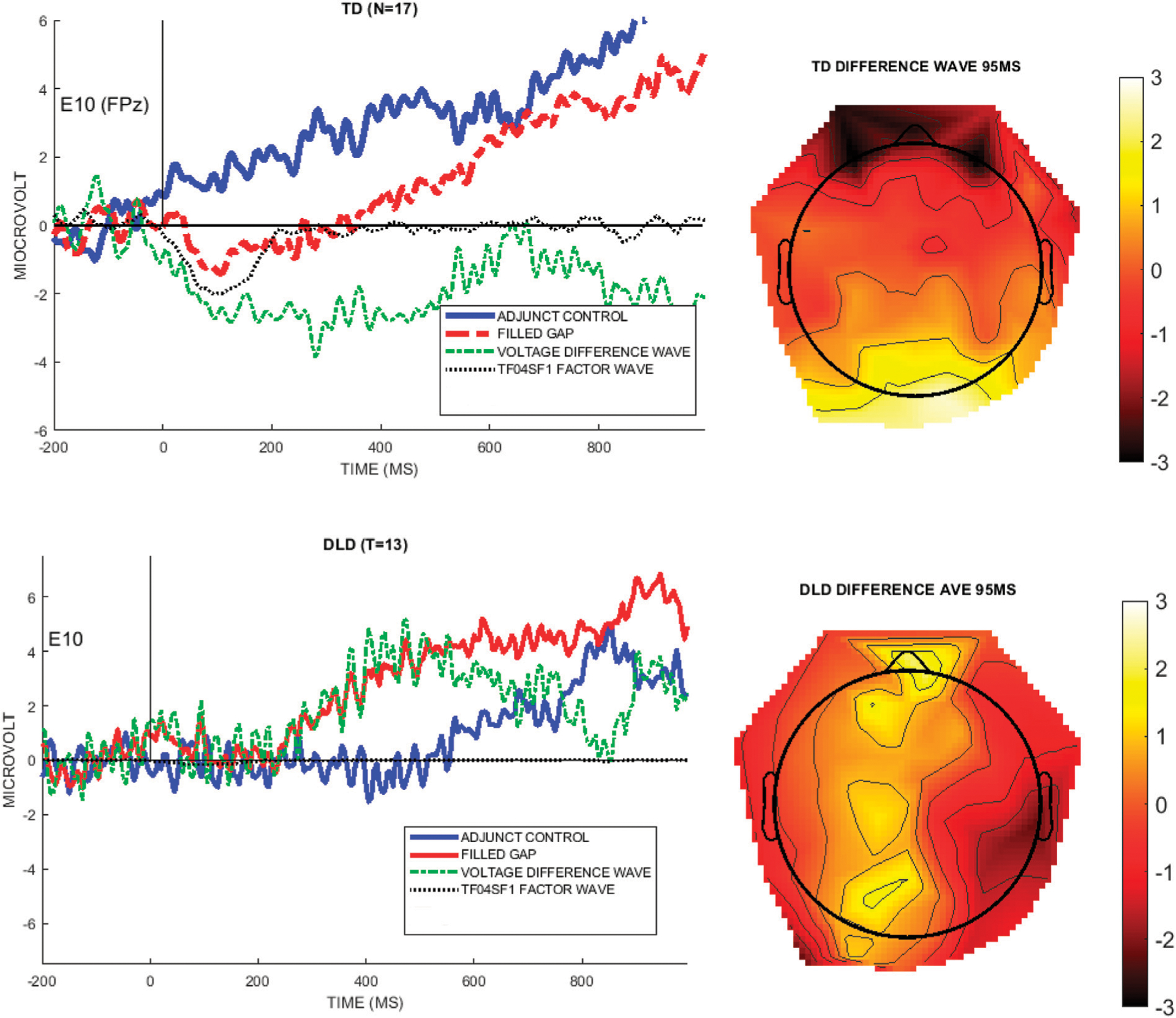
The left graphs show the voltage for each group at the channel with the highest weighting for the EAN component TF04SF1 (E10/FPz). The right images are topographical plots of the raw voltage difference between the Adjunct and Filled gap conditions at the peak latency of TF04 (95 ms), for each group.

To verify that the PCA/ICA factor matched the effect seen in raw data, we compared the EAN (TF04SF1) wave to the difference wave obtained from the raw voltage data, illustrated by the electrode where this PCA/ICA component was largest, specifically, E10 (FPz). Figure 7 shows the mean voltage waveforms for the control condition, filled gap condition, and the difference waveform, with the EAN (TF04F1) factor waveform overlaid (black dotted line), and illustrates that the temporal-spatial factor models the early negativity in the undecomposed voltage data.

#### 3.2.3 Voltage Analysis Constrained by the Early Bilateral Anterior Negativity (TF04SF1) Component

**FIGURE 8 |:**
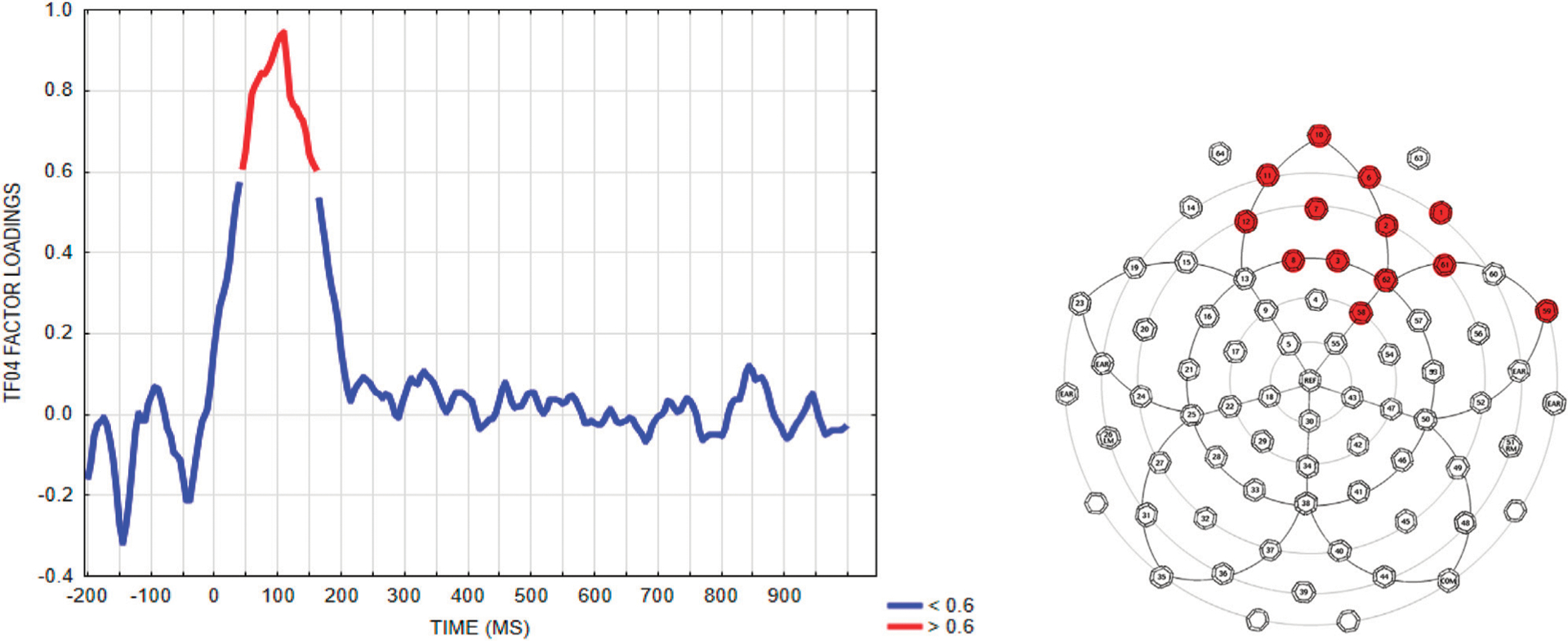
Time samples and electrodes exceeding 0.6 factor loadings selected from the EAN (TF04SF1) component, for deriving a voltage measure from this time window and electrode region.

To analyze the early anterior negativity using a more traditional approach, but that is guided by the PCA/ICA results, we used the method suggested in (Dien, 2012) by selecting a voltage “window” constrained by the PCA solution. We first selected a time window defined by the time points with EAN (TF04SF1) temporal factor loadings exceeding 0.6. This resulted in a 45–160 ms time window, as shown in the left graph of Figure 8, left panel. Next, an electrode region was selected by including electrodes that exceeded a factor loading of 0.6 for the EAN (TF04SF1) component. These were sites E1, E2, E3, E6, E7, E8, E10, E11, E12, E61, E62, E58, E59, as shown in Figure 8, right panel.^1^

**FIGURE 9 |:**
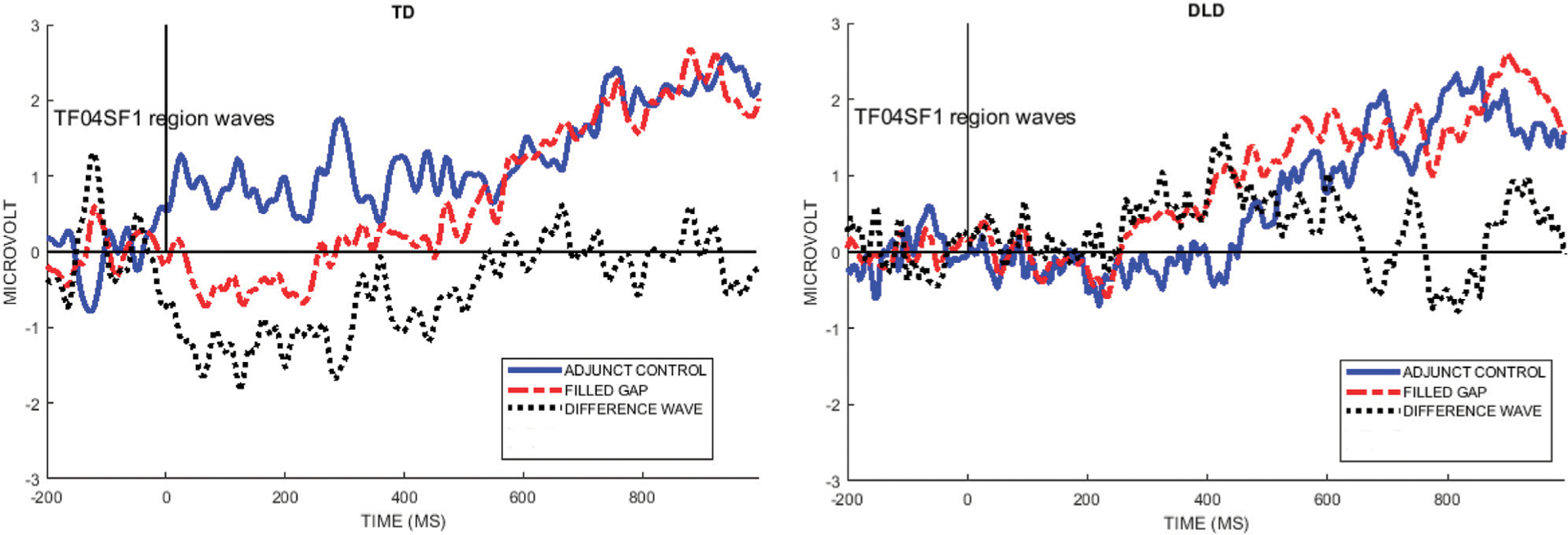
EAN (voltage-ERP); mean voltage waveforms for the EAN-region by group.

This time/space voltage construct, derived from the temporal and spatial weighting of the PCA/ICA-component TF04SF1, will be labeled “EAN (voltage-ERP)”, to express that it derives from the temporo-spatial PCA factor, and represents the same effect (but in unweighted voltage space) as the PCA factor “EAN (TF04SF1).” The mean waveform of the region consisting of these 13 sites is shown per condition and group in Figure 9.

The mean voltage for the 45–160 ms time-window and electrode region for each participant and trial in the filled gap and control condition were used as the dependent measures in mixed model statistical analysis.

We performed a linear mixed-effects analysis using R (version 4.1.2) and *lme4* (version 1.1.27; Bates et al., 2015). The input data were the voltage values for each of the trials remaining in each condition after artifact correction, thus varying by subject and cell. We started with the maximal random effects structure and gradually reduced the random effects until the model converged. The fixed effects were Group (typical vs. DLD), Condition (control vs. filled gap) and their interaction. The model converged when we included Subject as a random intercept. We report the model’s standardized coefficients after constructing orthogonal contrasts for the fixed effects, using the model parameters function from the *parameters* package (Lüdecke, Ben-Shachar, Patil, and Makowski, 2020), cf. Table 3.

**FIGURE 10 |:**
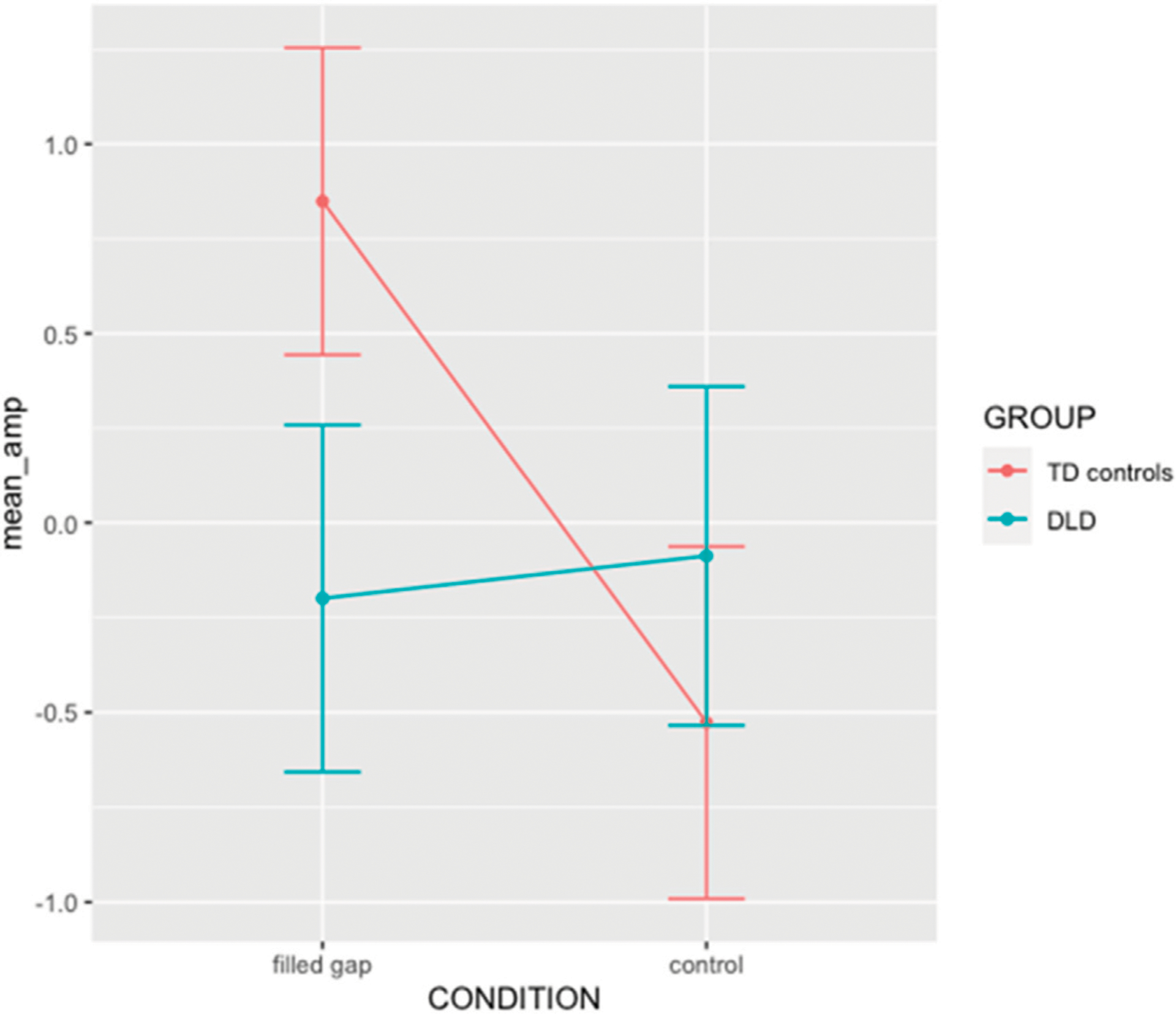
Interaction plot for the 2×2 design GROUP × Condition interaction in TF04SF1. Error bar indicate standard error.

The overall effect of each factor was estimated with Type III Wald chi-square tests using the *Anova* function from the *car* package (Fox & Weisberg, 2019). The effect was non-significant for Group (χ^2^ = 0.865, *p* = 0.352), Condition (χ^2^ = 1.872, *p* = 0.171), and the interaction term was not significant (χ^2^ = 3.619, *p* = 0.057); cf. Figure 10.

Based on our previous findings for adults using the same paradigm (Hestvik et al., 2012, 2007) and findings that typically developing children exhibit adult-like brain responses to syntactic violations from around 7 years of age (Hahne et al., 2004), we conducted the experiment with the expectation that the TD group should exhibit an eLAN or a similar early anterior negativity. We also expected the experiment to reveal whether DLD children did or did not show this effect. As shown in the interaction plot in Figure 10, the expectation for the TD group appears to be borne out, while the DLD group shows a flat response.

We therefore set orthogonal contrasts to compare filled gap vs. control in each level of Group. The standardized model coefficients corresponding to the simple effects revealed a significant effect of Condition for the TD control group (b = 0.14, 95% CI = [0.03, 0.26], t = 2.49, *p* = 0.013) but not for the DLD group (b = −0.02, 95% CI = [−0.15, 0.11], t = −0.35, *p* = 0.723). This bears out the prediction that TD children should show an eLAN-like brain response to the prediction violation and reveals that the DLD children do not respond to the violation in this early time window.

#### 3.2.4 Exploratory Analysis of Developmental Language Disorder Late Effect

Although the temporo-spatial factor TF03SF1 did not contain a statistically significant difference between the filled gap and control condition in the factor score analysis, it was the only factor that showed a DLD-specific response to the filled gap. The effect was also visible as a late right-posterior negativity combined with an anterior positivity in the grand average undecomposed voltage data, with a separation of conditions roughly in the 500–700 ms time window, using an 84% confidence interval (cf. Figure 5). Given this, as well as previous literature that have reported observing N400 to filled gaps in DLD children (Fonteneau and van der Lely, 2008), we conducted an exploratory PCA analysis limited to the DLD children to ascertain whether there was evidence indicating differential processing of the filled gap and control condition in the brain response

**FIGURE 11 |:**
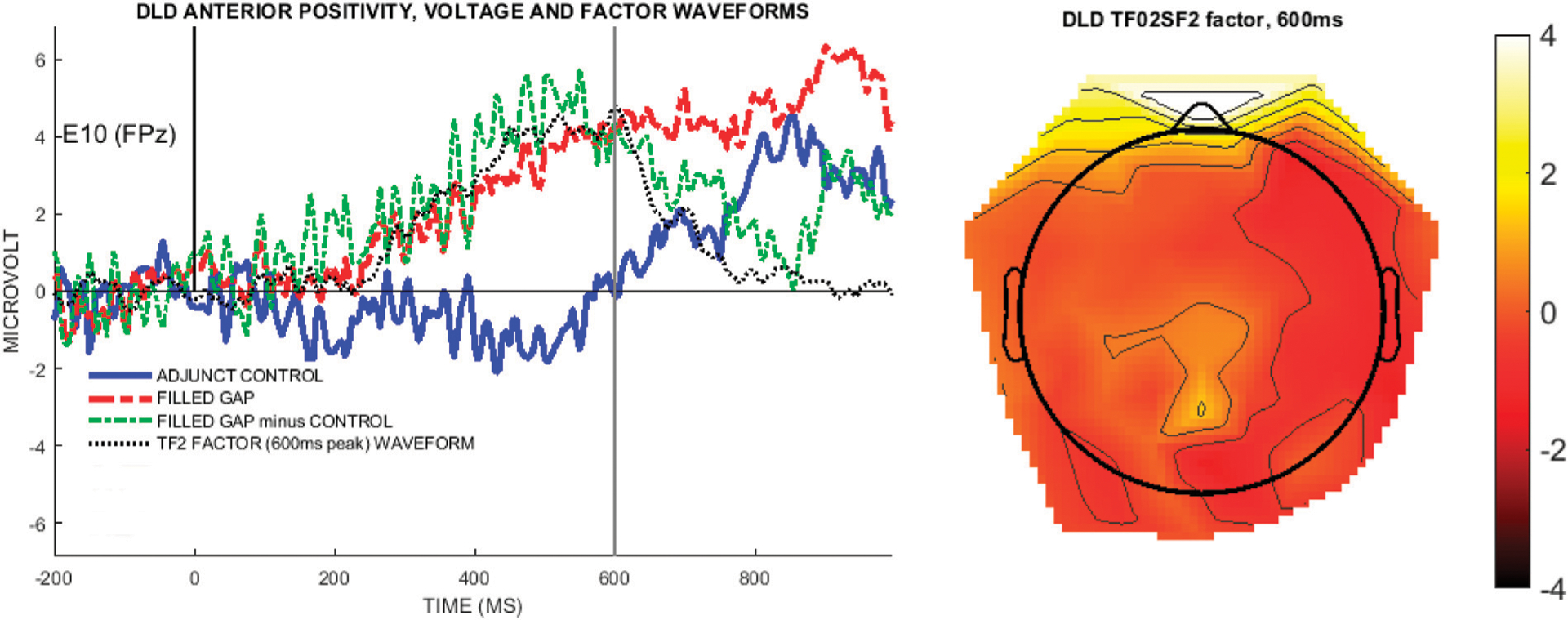
The left panel shows the temporal factor waveform derived from the voltage difference waveform (filled gap minus control), overlaid with the undecomposed voltage waveforms for the filled gap and control condition waveforms, for E10 (FPz), the peak positive channel in TF02SF2. The right topoplot shows the temporospatial factor TF02SF2 at 600 ms.

Using the difference wave (filled gap minus adjunct control) as input, the initial temporal PCA retained 12 factors, accounting for 89% of the total variance. The first three temporal factors each accounted for at least 5% of the variance and were selected for analysis. TF01 (980 ms) accounted for 39% variance, TF02 (600 ms) accounted for 26% of the variance, and TF03 (275 ms) account for 6% of the variance. Visual inspection indicated that TF02 in the DLD-only analysis, peaking at 600 ms, captured the same component as TF03 in the analysis with all children pooled, cf. Figure 5. The follow-up spatial ICA decomposition of each of the temporal factors retained 4 spatial factors for each temporal factor, based on the criteria used above. These combined temporo-spatial factors accounted for 70% of the DLD data. Among its spatial subfactors, TF02SF2 had the largest factor loadings (mean factor score = 4.8, SD = 8.5). The voltage waveforms for the electrode showing peak positivity for TF02SF2 are shown in Figure 11, along with the overall TF2SF2 topography at 600 ms.

Temporal factor loadings exceeding 0.6 were used to construct a time window of 345–635 ms for analysis of the voltage data. All spatial factor loadings were below 0.6; we therefore simply computed the mean voltage in the time window for the peak positive channels (E10/FPz) and tested whether it was different from zero with a *t*-test. This anterior positivity was not significantly different from zero (mean = 4 μV, standard error = 2.33, t(12) = 1.72, *p* = 0.11).

**FIGURE 12 |:**
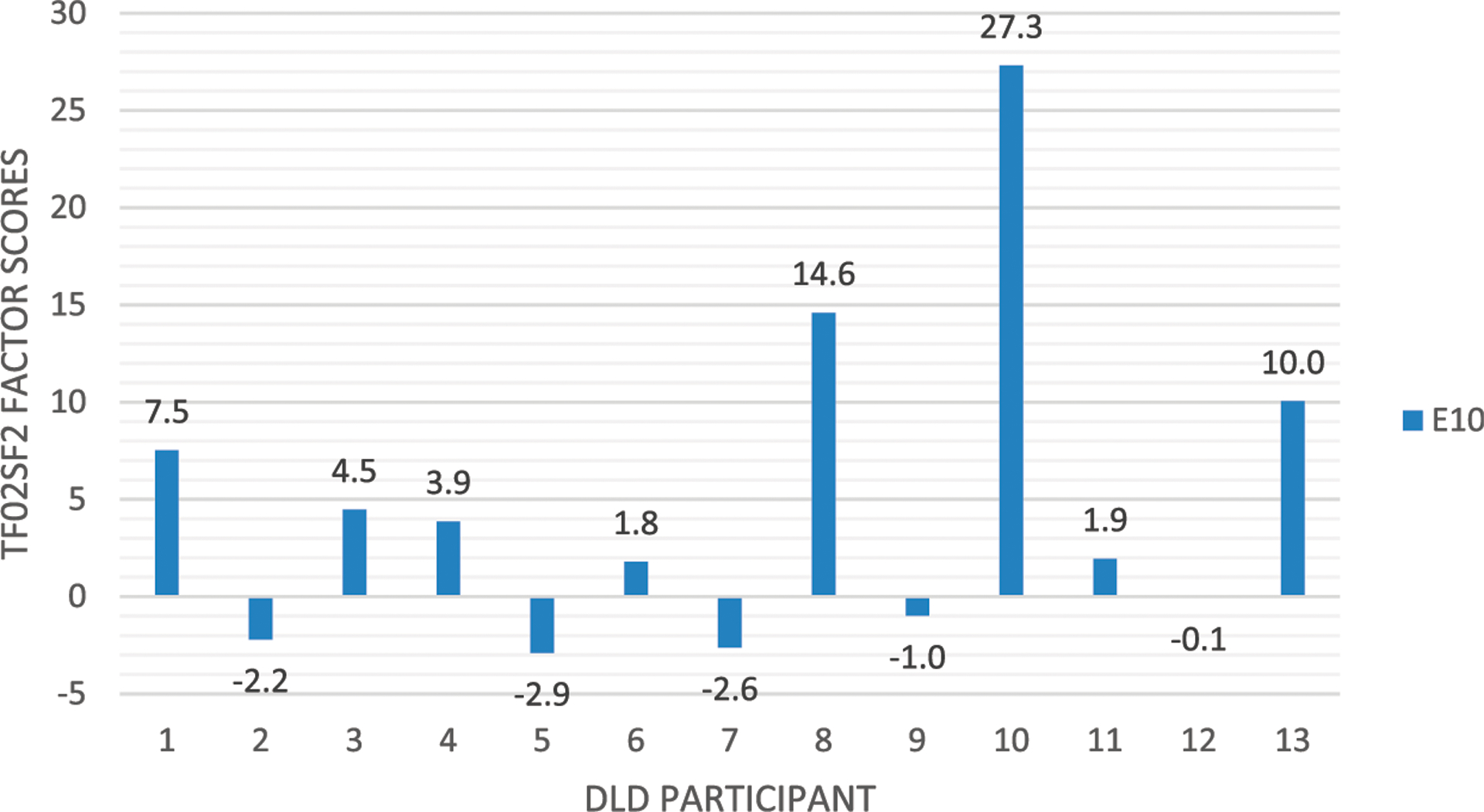
Distribution of individual factor scores (representing the strength to which the individual participant contributed to the late ERP response to the filled gap).

The individual participants’ mean factor scores (expressing the experimental effect in TF02SF2) are shown in Figure 12 (following practice recommended in Rousselet et al. (2016)). This reveals heterogeneity in brain responses, suggesting individual differences among DLD children in how their parser responds to the stimuli.

## 4 DISCUSSION

The aim of this study was to measure whether children with DLD predict gap-positions after encountering fillers, in comparison to their typically developing peers. The current findings revealed that when typically developing children listen to relative clauses like “the zebra that the hippo kissed…”, they generate the expectation that there should be no direct object after the verb (because it instead contains a gap). When this prediction is violated by an “unexpectedly filled gap,” this triggers an early anterior negativity after about 100 ms after encountering the acoustic signal of an unexpected noun phrase (the word “the”). This brain response is strikingly similar to the anterior negativity observed in the same paradigm with adults (Hestvik et al., 2012, 2007) and suggests that 9–12 year old children with typical development are already showing mature patterns of sentence processing, at least for these structures; the same was found by Hahne et al. (2004). In contrast, our data suggest that children with DLD are not processing these structures in a mature fashion, and, in fact, exhibit a complete absence of a filled gap response. We interpret this to mean that children with DLD do not make filler-gap predictions during sentence comprehension. We next discuss several questions arising from this finding.

### 4.1 Why Does Early AN Provide Evidence of Prediction?

Why does early AN reflect prediction specifically, rather than an integration effect? As noted in the literature, the same ERP pattern can reflect both integration effects and prediction effects (Mantegna et al., 2019). We adopt the view in Dikker et al. (2009) that the earliness of the eLAN itself is a sign of prediction (see also Lau et al., 2006). It is early because top-down grammatical expectations translate into sensory-level predictions of phonetic form (DeLong et al., 2014a; DeLong et al., 2014b; DeLong et al., 2019; Delong et al., 2021). Specifically, a filler predicts a verb phrase with an absent NP. This prediction can be viewed as resulting in pre-activation of a hypothesized parse tree with no NP after the verb. When the parser encounters “the” which indeed introduces a NP, this phonetic signal is therefore highly unexpected. The salience of this phonetic signal plausibly generates a clear surprise response for several reasons. First, the definite determiner is the most frequent word in English (Aiden et al., 2014). Second, it is phonetically unusual, as one of only a handful of function words starting with the voiced dental fricative [ð]. The early nature of the filled gap response is also consistent with recent findings that the brain responds to words around 50 ms after acoustic information is processed (MacGregor et al., 2012). Donhauser and Baillet (2020) using auditory stimuli also found that higher level grammatical predictions translate into predictions at the phonetic level. If a filled gap were to be introduced by a determiner-less NP (such as the bare plural “camels”), this should give rise to a later response, because there is no unique phonetic signal of a bare plural NP. We have examined this prediction elsewhere (Bradley and Hestvik, 2010).

### 4.2 Lateralization of the Early AN

We observed a bilateral early anterior negativity that was slightly larger over the right than the left sites. Several other studies have also found bilateral early anterior negativity instead of eLAN to syntactic violations in adults (Kessler, 2003; Kessler et al., 2004; Pakulak and Neville, 2011) as well as in children (Sabisch et al., 2009). We interpret the bilateral anterior negativity in our study as functionally equivalent to the eLAN, indicating surprisal for an unexpected syntactic category. We do not assume a strict mapping between neurocognitive processes and the specific ERP latency and topography, but rather that there is a family of ERP responses indicating syntactic processing and syntactic anomaly detection. Alternatively, the eLAN may be bilaterally distributed and the finding of asymmetry is related to other factors that modulate the topography. Shafer et al. (2000) observed an attenuated frontal positivity over left sites time-locked to the onset of grammatical utterances that started with “the” for children with DLD compared to those with TD. In addition, processing of the right frontal sites was enhanced in children with DLD. This pattern suggests an alternative processing route that engages right hemisphere sites. It will be important in future studies to explore such hemispheric differences in processing both grammatical and ungrammatical sentences and in relation to DLD.

### 4.3 Relationship Between Prediction Impairment and Comprehension

Our behavioral data did not suggest a difference in comprehension between TD and DLD conditional on gap-filling. If children with DLD fail to predict where a gap for a filler should be located, how can they interpret and understand such sentences? It has been suggested that these children interpret filler-gap sentences via alternative processing mechanisms, such as “direct semantic association” (Pickering and Barry, 1991). According to this model, the filler is associated directly with the argument structure of a verb without the syntactic mediation of a gap (Friedmann and Novogrodsky, 2007). If so, the filled gap NP might be analyzed as a referent that cannot be integrated into the argument structure of an already-saturated verb, which predicts a lexical/semantic integration violation and an N400 response (Frisch et al., 2004; Raettig et al., 2010).

Some indication supporting this idea is, as we have shown, that some DLD children did exhibit a later latency ERP effect. However, the observed late DLD ERP response to filled gaps did not reach significance, which could be due to the small sample size (*N* = 13), or be due due to individual differences among the children with DLD, as such heterogeneity has been observed in other studies (Shafer et al., 2007, Shafer et al., 2011).

Behavioral support for the idea that the DLD children interpret filler-gap sentences via alternative routes comes from our results of the comprehension question part of the current experiment. The children were tasked with interpreting grammatical filler-gap stimuli and grammatical filler-gap Wh-questions about the stimuli. In this task, we only observed a main effect of group, such that DLD children had an overall 8% lower accuracy. Crucially, there was no interaction between the sentence type of the stimulus sentence and group: The DLD children exhibit the same accuracy pattern for sentences with filler-gap dependencies vs. no filler-gap dependency. For example, filler-gap sentences were harder to answer correctly than non-filler gap stimulus sentences for both groups. If children with DLD failed to compute the meaning of sentences with filler-gap dependencies, they should exhibit significantly lower accuracy on object relative clause stimulus sentences than children with TD. Similarly, the DLD children exhibited the same pattern of accuracy as a function of whether the question itself contains an object-gap vs. a subject gap. Object gaps require the construction of a filler-gap dependency and they are typically harder to answer correctly than subject Wh-questions and yes/no-questions. Again, DLD children did not perform significantly worse on object Wh-questions vs. subject Wh-questions, than the TD children. These results provide an indication that that DLD children can calculate the meaning of sentences with filler-gap dependencies via alternative processing mechanisms (Friedmann and Novogrodsky, 2007).

### 4.4 Relationship Between Prediction and Language Acquisition

The “failure to predict” proposed here could play a key role in explaining why some children develop impaired grammatical knowledge. Recent theoretical work on typical language acquisition has emphasized the link between development of syntactic parsing and syntactic acquisition: children must “learn to parse” in order to analyze input and acquire syntax (Trueswell and Gleitman, 2007; Phillips and Ehrenhofer, 2014; Omaki and Lidz, 2015; Pozzan and Trueswell, 2015; Rabagliati et al., 2016). Current acquisition models also emphasize the reliance on error-signals tied to prediction (Dell et al., 2000, 2014; Montgomery and Evans, 2009). The developing child learns by adjusting the parser (probably, at an implicit level) in response to error signals. If children with DLD fail to predict and therefore fail to generate error signals, error-signal driven acquisition mechanisms will not succeed.

### 4.5 Why do Developmental Language Disorders Children Not Predict?

The current article does not address the underlying cause for the lack of prediction. One possible explanation lies in the lower verbal working memory resources often observed in DLD (Marton and Schwartz, 2003). Elsewhere, we have reported on the Sustained Anterior Negativity ERP as an index of working memory in filler-gap processing, which was elicited to the questions in the current study (Epstein et al., 2013). In their brain response to the comprehension questions, children with typical language development (TD) were expected to show a sustained anterior negativity, reflecting holding the Wh-word in memory until reaching the gap position (Fiebach et al., 2001; Phillips and Ehrenhofer 2014). Adults in Epstein et al. (2013) showed the predicted sustained anterior negativity, whereas children with TD showed a sustained positivity. Children with DLD showed no effect. This suggests that poor performance in long-distance dependencies in children with DLD may be related to low working memory capacity.

In Hestvik et al. (2012) we addressed the relationship between the filled gap response and working memory resources. We conducted a study with typically developed adults and examined whether these participants also exhibited a WM span modulation of the filled gap response. We found a bilateral early anterior negativity (AN) and a P600 to the filled gap, as well as an interaction with verbal memory span such that low span participants exhibit a delayed onset latency of the AN and P600 by about 200 ms (Hestvik et al., 2012). However, the adults with low memory span exhibited the same AN/P600 pattern as high-span listeners, unlike children with DLD who exhibited an absence of early anterior negativity. Therefore, low WM span typical adults do not model DLD children. It is therefore still unclear if reduced working memory explains the complete lack of a filled gap ERP effect in children with DLD, and the underlying cause of lack of prediction during sentence comprehension in this population requires further studies (Jones et al., 2021).

### 4.6 Limitation and Future Directions

A limitation of the current study is the relatively low number of DLD participants and consequently low statistical power for detecting true effects. This was in large part due to the challenges of recruiting and finding participants that meets the inclusion criteria, despite the reported high prevalence of 7% in the population (Leonard, 2017). While the absence of an early anterior negativity in the DLD group is clear, this makes the interpretation of the observed late ERP response in the DLD group suggestive at this time, and future studies with increased power are needed to replicate this effect and determine whether it generalizes to the population.

## 5 CONCLUSION

The current study revealed that children with Developmental Language Disorder (DLD) are not using the same neuro-parsing routines in processing long-distance dependencies as children with typical development (TD). Children with TD exhibited an early anterior negativity to a filled gap expectation violation in object relative clauses, which indicates predictive processing. Children with DLD show no similar early brain response, suggesting lack of predictive processing. The DLD children appear to still compute the meaning of relative clauses which suggests that they may use a variety of different strategies to process these sentences, despite their prediction impairment.

## Supplementary Material

stimuli.docx

## Figures and Tables

**TABLE 1 |:** Participant profiles with standard scores.

Group	Measure	Age	CELF-R	CELF-E	PPVT (−3 or −4)	TONI
DLD (N = 13)	Mean	10;1	79.92	76.38	85.54	98.23
	SD (months)	15 months	13.71	11.67	9.44	15.83
	Range	8;6–12;5	51–102	49–95	70–101	80–135
TD (N = 16*)	Mean	10;4	108.56	105.50	104.63	107.38
	SD (months)	12 months	12.13	13.42	12.15	12.12
	Range	8;5–12;3	88–125	89–133	86–129	90–130

* (One TD participant did not take the CELF and PPVT tests; but was judged to have normal language development by a licensed speech language pathologist. For this reason, we report N = 16 in this table, but the ERP data are based on N = 17.)

**TABLE 2 |:** Sentence types.

Type	Label	Example
Test	Filled gap	The zebra that the hippo kissed the camel on the nose ran far away
Control	Adjunct	The weekend that the hippo kissed the camel on the nose he ran far away
Fillers	Object Relative	The zebra that the hippo kissed on the nose ran far away
Fillers	Declarative	The zebra said that the hippo kissed the camel on the nose and ran far away
Fillers	Temporal	The cockatoo squawked at the peacock before cleaning its feathers

**TABLE 3 |:** Results of linear mixed model analysis.

Parameter	Coefficient	SE	95% CI	*t*	*p*
(Intercept)	−0.01	0.03	[−0.06, 0.04]	−0.38	0.701
Group	−0.05	0.06	[−0.16, 0.06]	−0.93	0.352
Condition	−0.06	0.04	[−0.15, 0.03]	−1.37	0.171
Group * Cond	0.17	0.09	[−0.01, 0.34]	1.90	0.057

**FIGURE A1 |:**
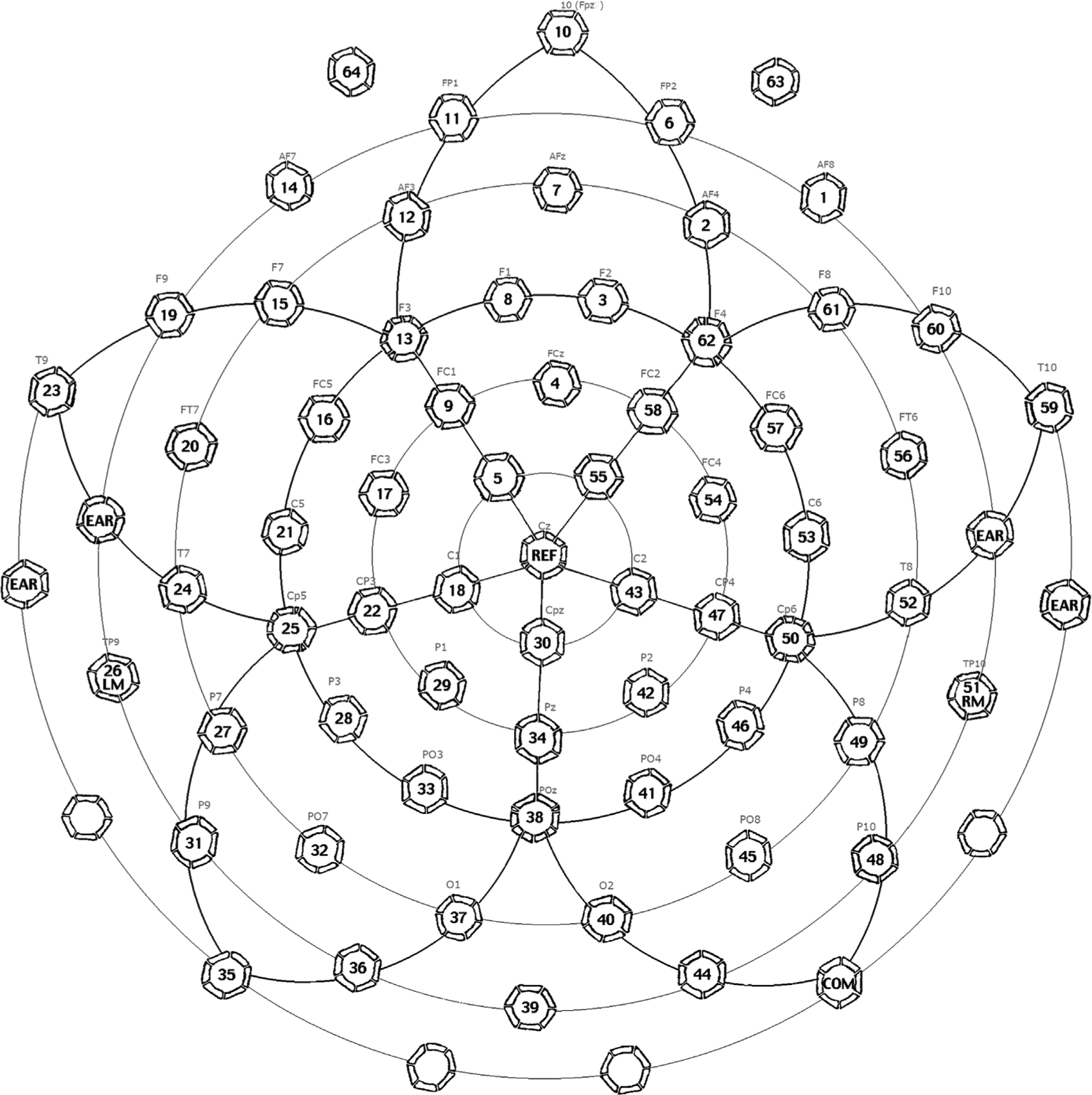
EGI GSN 64 v.2 montage with closest labels in the 10–10 labels.

## Data Availability

The raw data supporting the conclusions of this article is available at https://osf.io/m84jb/.

## HIGHLIGHTS

- Typically developing children exhibit a very early brain response to prediction error during sentence processing
- Developmental Language Disorder children do not exhibit this brain response
- The finding suggest that Developmental Language Disorder involves a prediction impairment
- Results are interpreted within a model of development that relates language acquisition to parsing development

## Footnotes

1 0.6 is an arbitrary treshhold but corresponds to a set of samples where the factor is highly weighted and delineates the effect in time and space.
