# Supplementary material for: Developmental Language Disorder as Syntactic Prediction Impairment
Source: Front Commun (Lausanne). Author manuscript; Available in PMC 2022 Mar 1. (PMC8887879; doi:10.3389/fcomm.2021.637585)
Supplement: stimuli.docx [file NIHMS1766712-supplement-stimuli_docx.docx]

Supplement: Stimuli

| CONDITION | Set | Script | Sentence Text | Comprehension Question | Q-type | Alt1 | Alt2 |
| --- | --- | --- | --- | --- | --- | --- | --- |
| FILLED GAP | 1 | A | The bear that the gorilla scared the tiger by accident went swimming in the pool. | Who did the gorilla scare | obQ+ | tiger | bear |
|  |  | B | The bear that the gorilla followed the tiger in the woods hid behind a tree. | Who followed the tiger | suQ+ | gorilla | bear |
|  | 2 | A | The bee that the ant rubbed the butterfly on the back dreamed about flowers. | Did the butterfly pinch the ant | ynQ+ | yes | no |
|  |  | B | The bee that the ant pinched the butterfly for no reason looked for a new home. | Who pinched the bee | suQ+ | noone | spider |
|  | 3 | A | The butterfly that the spider pinched the bee for no reason looked for a new home. | Who pinched the bee | suQ+ | spider | butterfly |
|  |  | B | The butterfly that the spider rubbed the bee on the back dreamed about flowers. | Who did the spider rub | obQ+ | bee | butterfly |
|  | 4 | A | The calf that the chicken tickled the lamb in the hay drank from a bowl. | Did you hear the word hay | yn2+ | yes | no |
|  |  | B | The calf that the chicken called the lamb in the meadow danced in a puddle. | Did the chicken call the lamb | ynQ+ | yes | no |
|  | 5 | A | The camel that the rhino watched the zebra from a distance peeked through the leaves. | Who did the rhino watch | obQ+ | zebra | camel |
|  |  | B | The camel that the rhino kissed the zebra on the nose ran far away. | Who kissed the zebra | suQ+ | rhino | camel |
|  | 6 | A | The chick that the eagle chased the seagull around the yard climbed on a rock. | Did the rooster chase the turkey | ynQ+ | yes | no |
|  |  | B | The chick that the eagle loved the seagull for years sat in the dust. | Did you hear the word years | yn2+ | yes | no |
|  | 7 | A | The chipmunk that the beaver startled the coyote near the log hopped up and down. | Who startled the raccoon | suQ+ | noone | beaver |
|  |  | B | The chipmunk that the beaver surprised the coyote in the woods rolled down the hill. | Who did the beaver surprise | obQ+ | chipmunk | raccoon |
|  | 8 | A | The dog that the cat patted the rabbit on the back napped in the grass. | Did the rabbit pat the cat | ynQ+ | yes | no |
|  |  | B | The dog that the cat touched the rabbit very carefully went to the playground. | Did you hear the word girl | yn2+ | yes | no |
|  | 9 | A | The dolphin that the panda poked the ape on the side laughed out loud. | Who poked the shark | suQ+ | panda | noone |
|  |  | B | The dolphin that the panda raced the ape to the shore blinked in the sun. | Who did the shark race | obQ+ | dolphin | noone |
|  | 10 | A | The donkey that the goat pushed the horse through the door fell in the mud. | Did you hear the word door | yn2+ | yes | no |
|  |  | B | The donkey that the goat kicked the horse in the field trotted into the barn. | Did the horse kick the goat | ynQ+ | yes | no |
|  | 11 | A | The duck that the leopard saw the cheetah in the morning floated in the pond. | Who did the fish see | obQ+ | noone | cheetah |
|  |  | B | The duck that the leopard bumped the cheetah from behind moved through the weeds. | Who bumped the chicken | suQ+ | leopard | noone |
|  | 12 | A | The duckling that the mouse called the turtle in the meadow danced in a puddle. | Did the lamb call the turtle | ynQ+ | yes | no |
|  |  | B | The duckling that the mouse tickled the turtle in the hay drank from a bowl. | Did you hear the word that | yn2+ | yes | no |
|  | 13 | A | The flamingo that the alligator tapped the elephant on the head ate breakfast early in the morning. | Who did the alligator tap | obQ+ | elephant | flamingo |
|  |  | B | The flamingo that the alligator heard the elephant in the dark stared at the moon. | Who liked the kitten | suQ+ | noone | alligator |
|  | 14 | A | The giraffe that the monkey greeted the kangaroo at the zoo jumped over the fence. | Did the monkey greet the kangaroo | ynQ+ | yes | no |
|  |  | B | The giraffe that the monkey took the kangaroo down the road came to the river. | Did you hear the word road | yn2+ | yes | no |
|  | 15 | A | The hen that the rooster loved the turkey for years sat in the dust. | Who did the rooster love | obQ+ | turkey | hen |
|  |  | B | The hen that the rooster chased the turkey around the yard climbed on a rock. | Who chased the rooster | suQ+ | noone | hen |
|  | 16 | A | The horse that the cow kicked the donkey in the field trotted into the barn. | Did the cow kiss the donkey | ynQ+ | yes | no |
|  |  | B | The horse that the cow pushed the donkey through the door fell in the mud. | Did you hear the word fence | yn2+ | yes | no |
|  | 17 | A | The kangaroo that the ostrich raced the giraffe down the road came to the river. | Who did the ostrich race | obQ+ | giraffe | kangaroo |
|  |  | B | The kangaroo that the ostrich greeted the giraffe at the zoo jumped over the fence. | Who greeted the giraffe | suQ+ | ostrich | kangaroo |
|  | 18 | A | The kitten that the bat visited the pig before breakfast crawled across the floor. | Did you hear the word breakfast | yn2+ | yes | no |
|  |  | B | The kitten that the bat liked the pig so much played in the garden. | Did the baby like the pig | ynQ+ | yes | no |
|  | 19 | A | The mouse that the hamster met the rat near the rose bushes rested after dinner. | Who did the snake meet | obQ+ | mouse | noone |
|  |  | B | The mouse that the hamster helped the rat in the afternoon slept in the sun. | Who helped the snail | suQ+ | noone | snake |
|  | 20 | A | The otter that the penguin smelled the walrus in the air slipped on the ice. | Did you hear the word summer | yn2+ | yes | no |
|  |  | B | The otter that the penguin hugged the walrus at bedtime snored during the night. | Did the penguin hug the walrus | ynQ+ | yes | no |
|  | 21 | A | The owl that the goose frightened the pigeons at night landed in the tree. | Who frightened the pigeon | suQ+ | goose | owl |
|  |  | B | The owl that the goose teased the pigeon all day flew through the air. | Who did the goose tease | obQ+ | owl | pigeon |
|  | 22 | A | The parrot that the cub teased the woodpecker all day flew through the air. | Did you hear the word robin | yn2+ | yes | no |
|  |  | B | The parrot that the cub frightened the woodpecker at night landed in the tree. | Did the pigeon frighten the goose | ynQ+ | yes | no |
|  | 23 | A | The pig that the puppy liked the kitten so much played in the garden. | Who did the puppy like | obQ+ | pig | dontknow |
|  |  | B | The pig that the puppy visited the kitten before breakfast crawled across the floor. | Who did the puppy visit | obQ+ | kitten | pig |
|  | 24 | A | The rabbit that the bird touched the dog very carefully went to the playground. | Did you hear the word month | yn2+ | yes | no |
|  |  | B | The rabbit that the bird patted the dog on the back napped in the grass. | Did the dog pat the bird | ynQ+ | yes | no |
|  | 25 | A | The reindeer that the otter hugged the guinea pig at bedtime snored during the night. | Who hugged the penguin | suQ+ | otter | noone |
|  |  | B | The reindeer that the otter smelled the guinea pig in the air slipped on the ice. | Who did the walrus smell | obQ+ | otter | noone |
|  | 26 | A | The skunk that the raccoon surprised the squirrel in the woods rolled down the hill. | Did you hear the word possum | yn2+ | yes | no |
|  |  | B | The skunk that the raccoon startled the squirrel near the log hopped up and down. | Did the squirrel startle the raccoon | ynQ+ | yes | no |
|  | 27 | A | The tiger that the lion followed the bear in the woods hid behind a tree. | Who followed the bear | suQ+ | lion | tiger |
|  |  | B | The tiger that the lion scared the bear by accident went swimming in the pool. | Who did the lion scare | obQ+ | bear | tiger |
|  | 28 | A | The toad that the snail helped the snake in the afternoon slept in the sun. | Did the snail help the snake | ynQ+ | yes | no |
|  |  | B | The toad that the snail met the snake near the rose bushes rested after dinner. | Did you hear the word day | yn2+ | yes | no |
|  | 29 | A | The turtle that the frog bumped the duck from behind moved through the weeds. | Who bumped the duck | suQ+ | frog | turtle |
|  |  | B | The turtle that the frog saw the duck in the morning floated in the pond. | Who did the frog see | obQ+ | duck | turtle |
|  | 30 | A | The whale that the seal took the shark to the shore blinked in the sun. | Did you hear the word dolphin | yn2+ | yes | no |
|  |  | B | The whale that the seal poked the shark on the side splashed in the waves. | Did the shark poke the seal | ynQ+ | yes | no |
|  | 31 | A | The wolf that the buffalo heard the crocodile in the dark stared at the moon. | Who heard the elephant | suQ+ | buffalo | noone |
|  |  | B | The wolf that the buffalo tapped the crocodile on the head ate breakfast early in the morning. | Did you hear the word mouth | yn2+ | yes | no |
|  | 32 | A | The zebra that the hippo kissed the camel on the nose ran far away. | Did the camel kiss the hippo | ynQ+ | yes | no |
|  |  | B | The zebra that the hippo watched the camel from a distance peeked through the leaves. | Did you hear the word distance | yn2+ | yes | no |
| ADJUNCT CONTROL | 1 | A | The afternoon that the gorilla scared the tiger by accident, he apologized. | Who did the gorilla scare | obQ+ | tiger | bear |
|  |  | B | The night that the gorilla followed the tiger in the woods, there was a storm. | Who followed the tiger | suQ+ | gorilla | bear |
|  | 2 | A | The afternoon that the ant rubbed the butterfly on the back, we were away. | Did the butterfly pinch the ant | ynQ+ | yes | no |
|  |  | B | The morning that the ant pinched the butterfly for no reason, it was raining . | Who pinched the bee | suQ+ | noone | butterfly |
|  | 3 | A | The summer that the spider pinched the bee for no reason, it was hot. | Who pinched the bee | suQ+ | spider | butterfly |
|  |  | B | The weekend that the spider rubbed the bee on the back, they got lost. | Who did the spider rub | obQ+ | bee | butterfly |
|  | 4 | A | The spring that the chicken tickled the lamb in the hay, they became friends. | Did you hear the word hay | yn2+ | yes | no |
|  |  | B | The morning that the chicken called the lamb in the meadow, it was thundering. | Did the chicken call the lamb | ynQ+ | yes | no |
|  | 5 | A | The month that the rhino watched the zebra from a distance, it was foggy. | Who did the rhino watch | obQ+ | zebra | camel |
|  |  | B | The year that the rhino kissed the zebra on the nose, they ran away. | Who kissed the zebra | suQ+ | rhino | camel |
|  | 6 | A | The year that the eagle chased the seagull around the yard, it was hot. | Did the rooster chase the turkey | ynQ+ | yes | no |
|  |  | B | The time that the eagle loved the seagull for years, I won't forget. | Did you hear the word years | yn2+ | yes | no |
|  | 7 | A | The moment that the beaver startled the coyote near the log, we screamed. | Who startled the raccoon | suQ+ | noone | noone |
|  |  | B | The moment that the beaver surprised the coyote in the woods, it started pouring. | Who did the beaver surprise | obQ+ | coyote | chipmunk |
|  | 8 | A | The second that the cat patted the rabbit on the back, the door opened. | Did the rabbit pat the cat | ynQ+ | yes | no |
|  |  | B | The day that the cat touched the rabbit very carefully, the farmer appeared. | Did you hear the word girl | yn2+ | yes | no |
|  | 9 | A | The day that the panda poked the ape on the side, they were swimming. | Who poked the shark | suQ+ | panda | noone |
|  |  | B | The summer that the panda raced the ape to the shore, it was sunny. | Who did the shark race | obQ+ | noone | dolphin |
|  | 10 | A | The morning that the goat pushed the horse through the door, there was a visitor. | Did you hear the word door | yn2+ | yes | no |
|  |  | B | The winter that the goat kicked the horse in the field, it was snowing. | Did the horse kick the goat | ynQ+ | yes | no |
|  | 11 | A | The day that the leopard saw the cheetah in the morning, there was a fire. | Who did the fish see | obQ+ | cheetah | noone |
|  |  | B | The afternoon that the leopard bumped the cheetah from behind, a fight started. | Who bumped the chicken | suQ+ | leopard | noone |
|  | 12 | A | The month that the mouse called the turtle in the meadow, they danced. | Did the lamb call the turtle | ynQ+ | yes | no |
|  |  | B | The week that the mouse tickled the turtle in the hay, they fun. | Did you hear the word that | yn2+ | yes | no |
|  | 13 | A | The spring that the alligator tapped the elephant on the head, it was warm. | Who did the alligator tap | obQ+ | elephant | flamingo |
|  |  | B | The second that the alligator heard the elephant in the dark, he jumped. | Who liked the kitten | suQ+ | noone | alligator |
|  | 14 | A | The evening that the monkey greeted the kangaroo at the zoo, they a meeting. | Did the monkey greet the kangaroo | ynQ+ | yes | no |
|  |  | B | The week that the monkey took the kangaroo down the road, there was a contest. | Did you hear the word road | yn2+ | yes | no |
|  | 15 | A | The summer that the rooster loved the turkey for years, we all remember. | Who did the rooster love | obQ+ | turkey | hen |
|  |  | B | The evening that the rooster chased the turkey around the yard, there was a fight. | Who chased the rooster | suQ+ | turkey | noone |
|  | 16 | A | The moment that the cow kicked the donkey in the field, the horse walked in. | Did the cow kiss the donkey | ynQ+ | yes | no |
|  |  | B | The afternoon that the cow pushed the donkey through the door, they got sick. | Did you hear the word fence | yn2+ | yes | no |
|  | 17 | A | The winter that the ostrich raced the giraffe down the road, it was humid. | Who did the ostrich race | obQ+ | giraffe | kangaroo |
|  |  | B | The minute that the ostrich greeted the giraffe at the zoo, the zookeeper walked in. | Who greeted the giraffe | suQ+ | ostrich | kangaroo |
|  | 18 | A | The morning that the bat visited the pig before breakfast, he learned to walk. | Did you hear the word breakfast | yn2+ | yes | no |
|  |  | B | The year that the bat liked the pig so much, he hardly cried. | Did the baby like the pig | ynQ+ | yes | no |
|  | 19 | A | The minute that the hamster met the rat near the rose bushes, it was raining. | Who did the snake meet | obQ+ | mouse | noone |
|  |  | B | The minute that the hamster helped the rat in the afternoon, they shook hands. | Who helped the snail | suQ+ | noone | snake |
|  | 20 | A | The night that the penguin smelled the walrus in the air, it was windy. | Did you hear the word summer | yn2+ | yes | no |
|  |  | B | The evening that the penguin hugged the walrus at bedtime, they stayed up late. | Did the penguin hug the walrus | ynQ+ | yes | no |
|  | 21 | A | The weekend that the goose frightened the pigeon at night, they flew away. | Who frightened the pigeon | suQ+ | goose | owl |
|  |  | B | The month that the goose teased the pigeon all day, we came to help. | Who did the goose tease | obQ+ | owl | pigeon |
|  | 22 | A | The time that the cub teased the woodpecker all day, they were arguing. | Did you hear the word robin | yn2+ | yes | no |
|  |  | B | The month that the cub frightened the woodpecker at night, there was a hurricane. | Did the pigeon frighten the goose | ynQ+ | yes | no |
|  | 23 | A | The time that the puppy liked the kitten so much, they were playmates. | Who did the puppy like | obQ+ | kitten | baby |
|  |  | B | The day that the puppy visited the kitten before breakfast, it was cloudy. | Who did the puppy visit | obQ+ | kitten | pig |
|  | 24 | A | The second that the bird touched the dog very carefully, he yelped. | Did you hear the word month | yn2+ | yes | no |
|  |  | B | The moment that the bird patted the dog on the back, it flew away. | Did the dog pat the bird | ynQ+ | yes | no |
|  | 25 | A | The night that the otter hugged the guinea pig at bedtime, he was snoring. | Who hugged the penguin | suQ+ | otter | noone |
|  |  | B | The spring that the otter smelled the guinea pig in the air, it was warm. | Who did the walrus smell | obQ+ | otter | noone |
|  | 26 | A | The minute that the raccoon surprised the squirrel in the woods, we got angry. | Did you hear the word possum | yn2+ | yes | no |
|  |  | B | The summer that the raccoon startled the squirrel near the log, they were in a fight. | Did the squirrel startle the raccoon | ynQ+ | yes | no |
|  | 27 | A | The year that the lion followed the bear in the woods, they were hunting. | Who followed the bear | suQ+ | lion | tiger |
|  |  | B | The spring that the lion scared the bear by accident, it was freezing. | Who did the lion scare | obQ+ | bear | tiger |
|  | 28 | A | The winter that the snail helped the snake in the afternoon, they chatted. | Did the snail help the snake | ynQ+ | yes | no |
|  |  | B | The weekend that the snail met the snake near the rose bushes, they played. | Did you hear the word day | yn2+ | yes | no |
|  | 29 | A | The weekend that the frog bumped the duck from behind, there was a holiday. | Who bumped the duck | suQ+ | frog | turtle |
|  |  | B | The time that the frog saw the duck in the morning, we escaped. | Who did the frog see | obQ+ | duck | turtle |
|  | 30 | A | The evening that the seal took the shark to the shore, it was stormy. | Did you hear the word dolphin | yn2+ | yes | no |
|  |  | B | The winter that the seal poked the shark on the side, it was snowing. | Did the shark poke the seal | ynQ+ | yes | no |
|  | 31 | A | The week that the buffalo heard the crocodile in the dark, it was windy. | Who heard the elephant | suQ+ | buffalo | noone |
|  |  | B | The second that the buffalo tapped the crocodile on the head, it became quiet. | Did you hear the word mouth | yn2+ | yes | no |
|  | 32 | A | The week that the hippo kissed the camel on the nose, there was a party. | Did the camel kiss the hippo | ynQ+ | yes | no |
|  |  | B | The night that the hippo watched the camel from a distance, it was raining. | Did you hear the word distance | yn2+ | yes | no |
| DECLARATIVE FILLERS | 1 | A | The bear knew that the gorilla scared the tiger by accident and then went swimming in the pool. | Who did the gorilla scare | obQ+ | tiger | bear |
|  |  | B | The bear knew that the gorilla followed the tiger in the woods and then hid behind a tree. | Who followed the tiger | suQ+ | gorilla | bear |
|  | 2 | A | The bee knew that the ant rubbed the butterfly on the back and then dreamed about flowers. | Did the butterfly pinch the ant | ynQ+ | yes | no |
|  |  | B | The bee knew that the ant pinched the butterfly for no reason and then looked for a new home. | Who pinched the bee | suQ+ | noone | butterfly |
|  | 3 | A | The butterfly knew that the spider pinched the bee for no reason and then looked for a new home. | Who pinched the bee | suQ+ | spider | butterfly |
|  |  | B | The butterfly knew that the spider rubbed the bee on the back and then dreamed about flowers. | Who did the spider rub | obQ+ | bee | butterfly |
|  | 4 | A | The calf knew that the chicken tickled the lamb in the hay and then drank from a bowl. | Did you hear the word hay | yn2+ | yes | no |
|  |  | B | The calf knew that the chicken called the lamb in the meadow and then danced in a puddle. | Did the chicken call the lamb | ynQ+ | yes | no |
|  | 5 | A | The camel dreamt that the rhino watched the zebra from a distance and then peeked through the leaves. | Who did the rhino watch | obQ+ | zebra | camel |
|  |  | B | The camel dreamt that the rhino kissed the zebra on the nose and then ran far away. | Who kissed the zebra | suQ+ | rhino | camel |
|  | 6 | A | The chick dreamt that the eagle chased the seagull around the yard and then climbed on a rock. | Did the rooster chase the turkey | ynQ+ | yes | no |
|  |  | B | The chick dreamt that the eagle loved the seagull for years and then sat in the dust. | Did you hear the word years | yn2+ | yes | no |
|  | 7 | A | The chipmunk dreamt that the beaver startled the coyote near the log and then hopped up and down. | Who startled the raccoon | suQ+ | noone | beaver |
|  |  | B | The chipmunk dreamt that the beaver surprised the coyote in the woods and then rolled down the hill. | Who did the beaver surprise | obQ+ | coyote | raccoon |
|  | 8 | A | The dog dreamt that the cat patted the rabbit on the back and then napped in the grass. | Did the rabbit pat the cat | ynQ+ | yes | no |
|  |  | B | The dog dreamt that the cat touched the rabbit very carefully and then went to the playground. | Did you hear the word girl | yn2+ | yes | no |
|  | 9 | A | The dolphin heard that the panda poked the ape on the side and then laughed out loud. | Who poked the shark | suQ+ | panda | noone |
|  |  | B | The dolphin heard that the panda raced the ape to the shore and then laughed out loud. | Who did the shark race | obQ+ | dolphin | noone |
|  | 10 | A | The donkey heard that the goat pushed the horse through the door and then fell in the mud. | Did you hear the word door | yn2+ | yes | no |
|  |  | B | The donkey heard that the goat kicked the horse in the field and then trotted into the barn. | Did the horse kick the goat | ynQ+ | yes | no |
|  | 11 | A | The duck heard that the leopard saw the cheetah in the morning and then floated in the pond. | Who did the fish see | obQ+ | noone | cheetah |
|  |  | B | The duck heard that the leopard bumped the cheetah from behind and then moved through the weeds. | Who bumped the chicken | suQ+ | leopard | noone |
|  | 12 | A | The duckling heard that the mouse called the turtle in the meadow and then danced in a puddle. | Did the lamb call the turtle | ynQ+ | yes | no |
|  |  | B | The duckling heard that the mouse tickled the turtle in the hay and then drank from a bowl. | Did you hear the word that | yn2+ | yes | no |
|  | 13 | A | The flamingo hoped that the alligator tapped the elephant on the head and then ate breakfast early in the morning. | Who did the alligator tap | obQ+ | elephant | flamingo |
|  |  | B | The flamingo hoped that the alligator heard the elephant in the dark and then stared at the moon. | Who liked the kitten | suQ+ | noone | alligator |
|  | 14 | A | The giraffe hoped that the monkey greeted the kangaroo at the zoo and then jumped over the fence. | Did the monkey greet the kangaroo | ynQ+ | yes | no |
|  |  | B | The giraffe hoped that the monkey took the kangaroo down the road and then came to the river. | Did you hear the word road | yn2+ | yes | no |
|  | 15 | A | The hen hoped that the rooster loved the turkey for years and then sat in the dust. | Who did the rooster love | obQ+ | turkey | hen |
|  |  | B | The hen hoped that the rooster chased the turkey around the yard and then climbed on a rock. | Who chased the rooster | suQ+ | noone | hen |
|  | 16 | A | The horse hoped that the cow kicked the doneky in the field and then trotted into the barn. | Did the cow kiss the donkey | ynQ+ | yes | no |
|  |  | B | The horse hoped that the cow pushed the donkey through the door and then fell in the mud. | Did you hear the word fence | yn2+ | yes | no |
|  | 17 | A | The kangaroo said that the ostrich raced the giraffe down the road and then came to the river. | Who did the ostrich race | obQ+ | giraffe | kangaroo |
|  |  | B | The kangaroo said that the ostrich greeted the giraffe at the zoo and then jumped over the fence. | Who greeted the giraffe | suQ+ | ostrich | kangaroo |
|  | 18 | A | The kitten said that the bat visited the pig before breakfast and then crawled across the floor | Did you hear the word breakfast | yn2+ | yes | no |
|  |  | B | The kitten said that the bat liked the pig so much and then played in the garden. | Did the baby like the pig | ynQ+ | yes | no |
|  | 19 | A | The mouse said that the hamster met the rat near the rose bushes and then rested after dinner. | Who did the snake meet | obQ+ | mouse | noone |
|  |  | B | The mouse said that the hamster helped the rat in the afternoon and then slept in the sun. | Who helped the snail | suQ+ | noone | snake |
|  | 20 | A | The otter said that the penguin smelled the walrus in the air and then slipped on the ice. | Did you hear the word summer | yn2+ | yes | no |
|  |  | B | The otter said that the penguin hugged the walrus at bedtime and then snored during the night. | Did the penguin hug the walrus | ynQ+ | yes | no |
|  | 21 | A | The owl pretended that the goose frightened the pigeon at night and then landed in the tree. | Who frightened the pigeon | suQ+ | goose | owl |
|  |  | B | The owl pretended that the goose teased the pigeon all day and then flew through the air. | Who did the goose tease | obQ+ | owl | pigeon |
|  | 22 | A | The parrot pretended that the cub teased the woodpecker all day and then flew through the air. | Did you hear the word robin | yn2+ | yes | no |
|  |  | B | The parrot pretended that the cub frightened the woodpecker at night and then landed in the tree. | Did the pigeon frighten the goose | ynQ+ | yes | no |
|  | 23 | A | The pig pretended that the puppy liked the kitten so much and then played in the garden. | Who did the puppy like | obQ+ | kitten | pig |
|  |  | B | The pig pretended that the puppy visited the kitten before breakfast and then crawled across the floor. | Who did the puppy visit | obQ+ | kitten | pig |
|  | 24 | A | The rabbit pretended that the bird touched the dog very carefully and then went to the playground. | Did you hear the word month | yn2+ | yes | no |
|  |  | B | The rabbit pretended that the bird patted the dog on the back and then napped in the grass. | Did the dog pat the bird | ynQ+ | yes | no |
|  | 25 | A | The reindeer guessed that the otter hugged the guinea pig at bedtime and then snored during the night. | Who hugged the penguin | suQ+ | otter | noone |
|  |  | B | The reindeer guessed that the otter smelled the guinea pig in the air and then slipped on the ice. | Who did the walrus smell | obQ+ | otter | noone |
|  | 26 | A | The skunk guessed that the raccoon surprised the squirrel in the woods and then rolled down the hill. | Did you hear the word possum | yn2+ | yes | no |
|  |  | B | The skunk guessed that the raccoon startled the squirrel near the log and then hopped up and down | Did the squirrel startle the raccoon | ynQ+ | yes | no |
|  | 27 | A | The tiger guessed that the lion followed the bear in the woods and then hid behind a tree. | Who followed the bear | suQ+ | lion | tiger |
|  |  | B | The tiger guessed that the lion scared the bear by accident and then went swimming in the pool. | Who did the lion scare | obQ+ | bear | tiger |
|  | 28 | A | The toad guessed that the snail helped the snake in the afternoon and then slept in the sun. | Did the snail help the snake | ynQ+ | yes | no |
|  |  | B | The toad guessed that the snail met the snake near the rose bushes and then rested after dinner. | Did you hear the word day | yn2+ | yes | no |
|  | 29 | A | The turtle thought that the frog bumped the duck from behind and then moved through the weeds. | Who bumped the duck | suQ+ | frog | turtle |
|  |  | B | The turtle thought that the frog saw the duck in the morning and then floated in the pond. | Who did the frog see | obQ+ | duck | turtle |
|  | 30 | A | The whale thought that the seal took raced the shark to the shore and then blinked in the sun. | Did you hear the word dolphin | yn2+ | yes | no |
|  |  | B | The whale thought that the seal poked the shark on the side and then splashed in the waves. | Did the shark poke the seal | ynQ+ | yes | no |
|  | 31 | A | The wolf thought that the buffalo heard the crocodile in the dark and then stared at the moon. | Who heard the elephant | suQ+ | buffalo | noone |
|  |  | B | The wolf thought that the buffalo tapped the crocodile on the head and then ate breakfast early in the morning. | Did you hear the word mouth | yn2+ | yes | no |
|  | 32 | A | The zebra thought that the hippo kissed the camel on the nose and then ran far away. | Did the camel kiss the hippo | ynQ+ | yes | no |
|  |  | B | The zebra thought that the hippo watched the camel from a distance and then peeked through the leaves. | Did you hear the word distance | yn2+ | yes | no |
| RELATIVE CLAUSE FILLERS | 1 | A | The bear that the gorilla scared by accident went swimming in the pool. | Who did the gorilla scare | obQ+ | bear | tiger |
|  |  | B | The bear that the gorilla followed in the woods hid behind a tree. | Who followed the tiger | suQ+ | noone | gorilla |
|  | 2 | A | The bee that the ant rubbed on the back dreamed about flowers. | Did the butterfly pinch the ant | ynQ+ | yes | no |
|  |  | B | The bee that the ant pinched for no reason looked for a new home. | Who pinched the bee | suQ+ | ant | noone |
|  | 3 | A | The butterfly that the spider pinched for no reason looked for a new home. | Who pinched the bee | suQ+ | noone | spider |
|  |  | B | The butterfly that the spider rubbed on the back dreamed about flowers. | Who did the spider rub | obQ+ | butterfly | bee |
|  | 4 | A | The calf that the chicken tickled in the hay drank from a bowl. | Did you hear the word hay | yn2+ | yes | no |
|  |  | B | The calf that the chicken called in the meadow danced in a puddle. | Did the chicken call the lamb | ynQ+ | yes | no |
|  | 5 | A | The camel that the rhino watched from a distance peeked through the leaves. | Who did the rhino watch | obQ+ | camel | zebra |
|  |  | B | The camel that the rhino kissed on the nose ran far away. | Who kissed the zebra | suQ+ | rhino | noone |
|  | 6 | A | The chick that the eagle chased around the yard climbed on a rock. | Did the rooster chase the turkey | ynQ+ | yes | no |
|  |  | B | The chick that the eagle loved for years sat in the dust. | Did you hear the word years | yn2+ | yes | no |
|  | 7 | A | The chipmunk that the beaver startled near the log hopped up and down. | Who startled the raccoon | suQ+ | noone | noone |
|  |  | B | The chipmunk that the beaver surprised in the woods rolled down the hill. | Who did the beaver surprise | obQ+ | raccoon | chipmunk |
|  | 8 | A | The dog that the cat patted on the back napped in the grass. | Did the rabbit pat the cat | ynQ+ | yes | no |
|  |  | B | The dog that the cat touched very carefully went to the playground. | Did you hear the word girl | yn2+ | yes | no |
|  | 9 | A | The dolphin that the panda poked on the side laughed out loud. | Who poked the shark | suQ+ | panda | noone |
|  |  | B | The dolphin that the panda raced to the shore blinked in the sun. | Who did the shark race | obQ+ | noone | dolphin |
|  | 10 | A | The donkey that the goat pushed through the door fell in the mud. | Did you hear the word door | yn2+ | yes | no |
|  |  | B | The donkey that the goat kicked in the field trotted into the barn. | Did the horse kick the goat | ynQ+ | yes | no |
|  | 11 | A | The duck that the leopard saw in the morning floated in the pond. | Who did the fish see | obQ+ | cheetah | noone |
|  |  | B | The duck that the leopard bumped from behind moved through the weeds. | Who bumped the chicken | suQ+ | leopard | noone |
|  | 12 | A | The duckling that the mouse called in the meadow danced in a puddle. | Did the lamb call the turtle | ynQ+ | yes | no |
|  |  | B | The duckling that the mouse tickled in the hay drank from a bowl. | Did you hear the word that | yn2+ | yes | no |
|  | 13 | A | The flamingo that the alligator tapped on the head ate breakfast early in the morning. | Who did the alligator tap | obQ+ | flamingo | elephant |
|  |  | B | The flamingo that the alligator heard in the dark stared at the moon. | Who liked the kitten | suQ+ | alligator | noone |
|  | 14 | A | The giraffe that the monkey greeted at the zoo jumped over the fence. | Did the monkey greet the kangaroo | ynQ+ | yes | no |
|  |  | B | The giraffe that the monkey took down the road came to the river. | Did you hear the word road | yn2+ | yes | no |
|  | 15 | A | The hen that the rooster loved for years sat in the dust. | Who did the rooster love | obQ+ | hen | turkey |
|  |  | B | The hen that the rooster chased around the yard climbed on a rock. | Who chased the rooster | suQ+ | rooster | noone |
|  | 16 | A | The horse that the cow kicked in the field trotted into the barn. | Did the cow kiss the donkey | ynQ+ | yes | no |
|  |  | B | The horse that the cow pushed through the door fell in the mud. | Did you hear the word fence | yn2+ | yes | no |
|  | 17 | A | The kangaroo that the ostrich raced down the road came to the river. | Who did the ostrich race | obQ+ | kangaroo | giraffe |
|  |  | B | The kangaroo that the ostrich greeted at the zoo jumped over the fence. | Who greeted the giraffe | suQ+ | noone | ostrich |
|  | 18 | A | The kitten that the bat visited before breakfast crawled across the floor. | Did you hear the word breakfast | yn2+ | yes | no |
|  |  | B | The kitten that the bat liked so much played in the garden. | Did the baby like the pig | ynQ+ | yes | no |
|  | 19 | A | The mouse that the hamster met near the rose bushes rested after dinner. | Who did the snake meet | obQ+ | noone | mouse |
|  |  | B | The mouse that the hamster helped in the afternoon slept in the sun. | Who helped the snail | suQ+ | noone | snake |
|  | 20 | A | The otter that the penguin smelled in the air slipped on the ice. | Did you hear the word summer | yn2+ | yes | no |
|  |  | B | The otter that the penguin hugged at bedtime snored during the night. | Did the penguin hug the walrus | ynQ+ | yes | no |
|  | 21 | A | The owl that the goose frightened at night landed in the tree. | Who frightened the pigeon | suQ+ | goose | noone |
|  |  | B | The owl that the goose teased all day flew through the air. | Who did the goose tease | obQ+ | pigeon | owl |
|  | 22 | A | The parrot that the cub teased all day flew through the air. | Did you hear the word robin | yn2+ | yes | no |
|  |  | B | The parrot that the cub frightened at night landed in the tree. | Did the pigeon frighten the goose | ynQ+ | yes | no |
|  | 23 | A | The pig that the puppy liked so much played in the garden. | Who did the puppy like | obQ+ | pig | kitten |
|  |  | B | The pig that the puppy visited before breakfast crawled across the floor. | Who did the puppy visit | obQ+ | kitten | pig |
|  | 24 | A | The rabbit that the bird touched very carefully went to the playground. | Did you hear the word month | yn2+ | yes | no |
|  |  | B | The rabbit that the bird patted on the back napped in the grass. | Did the dog pat the bird | ynQ+ | yes | no |
|  | 25 | A | The reindeer that the otter hugged at bedtime snored during the night. | Who hugged the penguin | suQ+ | otter | noone |
|  |  | B | The reindeer that the otter smelled in the air slipped on the ice. | Who did the walrus smell | obQ+ | otter | noone |
|  | 26 | A | The skunk that the raccoon surprised in the woods rolled down the hill. | Did you hear the word possum | yn2+ | yes | no |
|  |  | B | The skunk that the raccoon startled near the log hopped up and down. | Did the squirrel startle the raccoon | ynQ+ | yes | no |
|  | 27 | A | The tiger that the lion followed in the woods hid behind a tree. | Who followed the bear | suQ+ | lion | noone |
|  |  | B | The tiger that the lion scared by accident went swimming in the pool. | Who did the lion scare | obQ+ | tiger | bear |
|  | 28 | A | The toad that the snail helped in the afternoon slept in the sun. | Did the snail help the snake | ynQ+ | yes | no |
|  |  | B | The toad that the snail met near the rose bushes rested after dinner. | Did you hear the word day | yn2+ | yes | no |
|  | 29 | A | The turtle that the frog bumped from behind moved through the weeds. | Who bumped the duck | suQ+ | frog | noone |
|  |  | B | The turtle that the frog saw in the morning floated in the pond. | Who did the frog see | obQ+ | turtle | duck |
|  | 30 | A | The whale that the seal took to the shore blinked in the sun. | Did you hear the word dolphin | yn2+ | yes | no |
|  |  | B | The whale that the seal poked on the side splashed in the waves. | Did the shark poke the seal | ynQ+ | yes | no |
|  | 31 | A | The wolf that the buffalo heard in the dark stared at the moon. | Who heard the elephant | suQ+ | buffalo | noone |
|  |  | B | The wolf that the buffalo tapped on the head ate breakfast early in the morning. | Did you hear the word mouth | yn2+ | yes | no |
|  | 32 | A | The zebra that the hippo kissed on the nose ran far away. | Did the camel kiss the hippo | ynQ+ | yes | no |
|  |  | B | The zebra that the hippo watched from a distance peeked through the leaves. | Did you hear the word distance | yn2+ | yes | no |
